# Longitudinal gut microbiome dynamics are associated with clinical outcome and toxicity during ibrutinib therapy

**DOI:** 10.1080/19490976.2026.2659397

**Published:** 2026-04-19

**Authors:** Nadine Morineau, Benoît Tessoulin, Thomas Guimard, Mathilde Papin, Antoine Roquilly, Steven Le Gouill, Emmanuel Montassier

**Affiliations:** a Department of Hematology, Centre Hospitalier Départemental Vendée, La Roche-sur-Yon, France; b Service d'hématologie, centre hospitalier universitaire (CHU) Hôtel-Dieu, Nantes, France; c Centre de recherche en cancérologie et immunologie intégrée Nantes Angers, Inserm, centre national de la recherche scientifique, université d'Angers, université de Nantes, Nantes, France; d Service Médecine Post-Urgence-Infectiologie, CHD Vendée, La Roche-sur-Yon, France; e Service des Urgences, Nantes Université, CHU Nantes, Nantes, France; f Nantes Université, CHU Nantes, INSERM, Center for Research in Transplantation and Translational Immunology UMR, Nantes, France; g CHU Nantes, INSERM, Nantes Université, Anesthésie Réanimation, Nantes, France; h Service d’hématologie, Institut Curie, Saint Cloud, France, université Versailles Saint-Quentin (UVSQ), France, Laboratoire d’Imagerie Translationnelle en Oncologie (LITO), U1288 Inserm/Institut Curie centre de recherche, Paris, France; i CHU Nantes, INSERM, Nantes Université, Service des Urgences, Nantes, France

**Keywords:** Gut microbiome, longitudinal trajectory, biomarker, shotgun metagenomics, ibrutinib, treatment response, B-cell malignancies, ibrutinib-associated diarrhea

## Abstract

Accumulating evidence indicates that the gut microbiome influences therapeutic efficacy and toxicity across cancer treatments; however, its longitudinal dynamics during targeted therapies remain poorly characterized. Here, we performed whole-genome shotgun metagenomic sequencing of 291 longitudinal stool samples collected over one year from 30 patients with hematologic malignancies treated with ibrutinib. Overall gut microbial diversity remained stable at the population level but exhibited markedly divergent temporal trajectories according to clinical outcome, with progressive recovery in responders and blunted or delayed restoration in non-responders. Longitudinal modeling revealed distinct species- and pathway-level microbial dynamics between patients with treatment response or nonresponse, including enrichment of saccharolytic, short-chain fatty acid–associated taxa and metabolic pathways in responders, and expansion of bile acid–modifying, proteolytic, and inflammation-associated microbial features in non-responders. Functional profiling further demonstrated opposing temporal trends in pathways related to carbohydrate fermentation, amino-acid metabolism, and secondary bile acid synthesis. In addition, both baseline microbiome composition and longitudinal remodeling were associated with the development of ibrutinib-associated diarrhea. Together, these findings reveal coordinated, outcome-specific remodeling of the gut microbiome during ibrutinib therapy and highlight longitudinal microbiome trajectories, rather than static baseline features, as potential biomarkers of treatment response and toxicity, as well as targets for microbiome-directed interventions. In conclusion, our findings highlight a potential role of gut microbiome dynamics in modulating response to BTK inhibition and support the need for larger, prospective studies to validate these observations.

## 1. Introduction

In recent years, the human gut microbiome has emerged as a central regulator of antitumor immunity. Foundational studies in both preclinical models and human cohorts have demonstrated that intestinal microbial composition can influence the efficacy and toxicity of multiple immune-based cancer therapies, including immune checkpoint blockade, hematopoietic stem cell transplantation, and chimeric antigen receptor (CAR) T-cell therapy.^1-7^ As the gut microbiome constitutes the largest and most continuous source of non-self antigens encountered by the host immune system, it plays a critical role in shaping systemic immune tone and modulating both innate and adaptive immune responses. Accordingly, the clinical activity and safety of modern immunotherapies are increasingly understood as the product of a dynamic tripartite interaction between the therapeutic agent, the host immune system, and the gut microbiome.

Despite the growing recognition of microbiome–immune interactions in oncology, the role of the gut microbiome in patients treated with ibrutinib has not been methodically studied through longitudinal metagenomics. Ibrutinib is a first-in-class, covalent Bruton tyrosine kinase (BTK) inhibitor that irreversibly binds BTK at cysteine-481, thereby suppressing downstream B-cell receptor signaling pathways essential for B-cell activation, proliferation, survival, and tissue homing.^8^^,^^9^ In addition to its on-target effects, ibrutinib inhibits several off-target kinases, including IL-2–inducible T-cell kinase (ITK), TEC, EGFR, and BMX, resulting in broader immunomodulatory effects such as altered T-cell differentiation and a bias toward Th1-polarized immune responses.^10^^,^^11^ Through these combined mechanisms, ibrutinib not only exerts direct antitumor activity but also reshapes the immune landscape in treated patients.

Clinically, ibrutinib is approved for the treatment of multiple B-cell malignancies, including chronic lymphocytic leukemia (CLL), small lymphocytic lymphoma, mantle cell lymphoma, Waldenström macroglobulinemia, and marginal zone lymphoma, across both frontline and relapsed or refractory settings.^12-14^ Although ibrutinib has markedly improved patient outcomes, clinical responses remain heterogeneous, and treatment is frequently complicated by adverse events such as infections, diarrhea, cardiovascular toxicity, and immune dysregulation. In the largest retrospective multicenter study to date involving 616 patients with CLL, 41% discontinued ibrutinib therapy, with a median time to discontinuation of only seven months.^15^ These observations highlight a substantial unmet need to better understand the biological determinants of both treatment response and intolerance.

Given the established role of the gut microbiome in regulating immune activation, infection susceptibility, and systemic inflammatory tone, microbiome–drug–immune interactions may represent an unrecognized contributor to the variability in clinical outcomes observed with ibrutinib therapy. However, this dimension has not yet been investigated. In this study, we examined the impact of ibrutinib treatment on the intestinal microbiome and assessed whether specific microbial signatures were associated with therapeutic response and the development of gastrointestinal toxicity, particularly diarrhea. By integrating longitudinal gut microbiome profiling with detailed clinical endpoints, we provide the first evidence linking ibrutinib therapy to distinct patterns of microbiome composition and function.

## 2. Methods

### 2.1. Study design

This prospective longitudinal study included a convenient sample of 30 adult patients diagnosed with chronic lymphocytic leukemia, mantle cell lymphoma, or Waldenström macroglobulinemia who received ibrutinib therapy at La Roche-sur-Yon Hospital and Nantes University Hospital between February 2020 and May 2023. Patients received ibrutinib according to standard clinical practice and approved indications for their underlying B-cell malignancy. Ibrutinib was administered as continuous oral therapy at standard doses (typically 420 mg once daily for chronic lymphocytic leukemia and Waldenström macroglobulinemia and 560 mg once daily for mantle cell lymphoma). All patients in this cohort received ibrutinib as monotherapy. Exclusion criteria included a history of uncontrolled colitis before treatment initiation, known inflammatory bowel disease, probiotic consumption during the inclusion period, or inability or unwillingness to provide written informed consent. During follow-up, stool samples were prospectively and repeatedly collected from each participant at up to 12 predefined time points: before treatment initiation (baseline) and at days 15, 30, 45, 60, 75, 90, 120, 180, 240, 300, and 360 after starting therapy. This design enabled dense longitudinal sampling over one year of treatment and constitutes, to our knowledge, the most extensive fecal sampling framework reported to date for patients receiving ibrutinib.

Clinical and epidemiologic data were extracted from medical records and included age, sex, body mass index (BMI), Eastern Cooperative Oncology Group (ECOG) performance status, treatment response, gastrointestinal adverse events, number of prior lines of oncologic therapy, and systemic antibiotic exposure during the year preceding inclusion. Eligible participants were adults (≥18 years), not under legal guardianship, and receiving targeted therapy with ibrutinib. Exclusion criteria comprised a history of uncontrolled colitis before treatment initiation, known inflammatory bowel disease, or inability or unwillingness to provide written informed consent.

Clinical response to ibrutinib was defined according to standard disease-specific response criteria assessed during follow-up.^16^ Patients achieving a complete response, partial response, or sustained disease control without progression were classified as responders. Patients with primary refractory disease, disease progression, treatment discontinuation due to lack of efficacy, or death during follow-up were classified as non-responders.

All participants provided written informed consent prior to inclusion in the COLMI study and did not receive financial compensation. The acquisition and analysis of clinical data and biological specimens were approved by the Comité de Protection des Personnes Sud-Méditerranée II (protocol 218 C 21; approval date: June 13, 2018). The study was registered on ClinicalTrials.gov (NCT03569137).

### 2.2. Fecal sample collection

Fecal samples were collected from both inpatients and outpatients using the DNA/RNA Shield Fecal Collection Kit according to the manufacturer’s instructions. Samples were immediately stored at −80 °C upon receipt and maintained under frozen conditions until DNA extraction. At both participating centers, stool specimens were collected longitudinally at predefined intervals encompassing periods before, during, and after initiation of ibrutinib therapy.

### 2.3. DNA extraction and metagenome sequencing

All stool samples from La Roche-sur-Yon Hospital and Nantes University Hospital were processed centrally for DNA extraction at the GenoBird Platform (Nantes University).^17^ Bacterial genomic DNA was extracted using the QIAamp Fast DNA Stool Mini Kit (Qiagen), following the manufacturer’s protocol with the addition of an intensive bead-beating lysis step to enhance cell disruption. Briefly, samples were subjected to mechanical lysis using a 3.2-mm steel bead in combination with zirconium beads. Shotgun metagenomic sequencing libraries were prepared using the Illumina DNA Preparation Kit (Illumina; catalog no. 20060060). Library quality and fragment size distribution were assessed using an Agilent High Sensitivity D1000 ScreenTape assay (Agilent; catalog no. 5067-5584). Libraries were pooled at equimolar concentrations and sequenced on an Illumina NovaSeq X Series 25B platform.

### 2.4. Microbiome sequencing data processing

Microbiome sequencing and analysis were performed as previous described.^17^^,^^18^ Sequencing reads were demultiplexed according to sample-specific barcodes. All downstream bioinformatic analyses were conducted centrally at Nantes University. Taxonomic profiling was performed using MetaPhlAn v4.0, and functional profiling was carried out using HUMAnN3 v3.0, following recommended default parameters.^19^^,^^20^

### 2.5. Bioinformatics and statistical analysis

#### 2.5.1. Diversity analysis

Microbial alpha diversity was quantified for each sample using the Shannon diversity index computed on rarefied count data with the phyloseq R package.^21^ Longitudinal changes in Shannon diversity and their association with clinical outcome (responders vs non-responders) were evaluated using linear mixed-effects models.^22^ Because each participant contributed repeated measurements across predefined time points, subject-specific random intercepts were included to account for within-subject correlation. Longitudinal microbiome responses to therapeutic interventions are often characterized by non-linear temporal dynamics, reflecting phases of treatment-associated perturbation, transient microbial instability, and potential delayed ecological recovery or adaptation.^23^ To flexibly capture non-linear temporal trends, time since treatment initiation (in days) was modeled using a natural cubic spline with four degrees of freedom. The full model included clinical outcome, spline-modeled time, and their interaction, as well as covariates known or suspected to influence gut microbiota diversity, including sex, age, BMI, antibiotic exposure during the preceding year, prior chemotherapy, and cohort. Models were fitted using restricted maximum likelihood estimation. Statistical significance was assessed using Satterthwaite-approximated degrees of freedom, with a two-sided *α* level of 0.05.

Overall microbial community composition was assessed using Bray–Curtis dissimilarity calculated from all longitudinal samples. Bray–Curtis dissimilarity was selected because it is widely used for shotgun metagenomic community profiles and provides a robust measure of compositional differences based on species abundances. In contrast, phylogeny-aware beta-diversity metrics such as UniFrac require a consistent phylogenetic tree derived from homologous marker sequences, which is not directly available for species abundance tables generated from shotgun metagenomic profiling approaches such as MetaPhlAn. To model non-linear temporal dynamics in community structure, a natural cubic spline basis (four degrees of freedom) was generated for the time variable, yielding four orthogonal spline components. These spline terms were included to test whether microbial community trajectories differed over time according to clinical outcome. Permutational multivariate analysis of variance (PERMANOVA) was performed using the adonis2 function in the vegan package, with permutations restricted within subjects to account for repeated measurements.^24^ Marginal effects were evaluated using the by = “margin” option. The model included clinical outcome, its interaction with spline-modeled time, and the same covariates used for alpha-diversity analyses. Statistical significance was assessed using 999 permutations. Analyses were performed in R (version 4.4.1) using the vegan, splines2, lme4, lmerTest, emmeans, and ggplot2 packages.^22^^,^^24-27^ Moreover, homogeneity of multivariate dispersion between outcome groups was evaluated using the betadisper function from the vegan R package.^24^

#### 2.5.2. Differential abundance analysis at baseline and longitudinal modeling of microbial trajectories

To identify bacterial species differentially abundant at baseline between responders and non-responders to ibrutinib, compositional differential abundance analysis was performed using ANCOM-BC2 at the species level.^28^ Microbial abundance tables were aligned with clinical metadata by sample identifier, retaining samples present in both datasets. Analyses were restricted to species present in at least 5% of samples and with a mean relative abundance ≥0.001 to reduce the influence of rare taxa.^29^ Differential abundance was modeled using bias-corrected log-linear mixed-effects models that account for compositionality, structural zeros, and within-subject correlation. Models were adjusted for age, sex, BMI, cohort, and prior exposure to antibiotics and chemotherapy. Multiple testing correction was performed using the false discovery rate (FDR) approach.^30^

To characterize longitudinal changes in gut microbiome composition and their association with clinical outcome, we applied a trajectory-based modeling framework adapted for longitudinal microbiome data.^31^ We used the R package Fido, which implements a Bayesian multinomial logistic normal regression model (Pibble).^32^ In this framework, statistical inference is based on posterior distributions of model coefficients rather than *p*-values, and classical multiple testing correction procedures such as false discovery rate adjustment are not applied. Instead, differential associations are evaluated using posterior summaries, including credible intervals and posterior probability estimates, within a joint multivariate model that accounts for covariance between microbial features. Analyses were conducted at the species level using relative abundance profiles derived from shotgun metagenomic sequencing. Relative abundances were analyzed on the log-ratio scale to account for the compositional nature of microbiome data.^33^ Regression models incorporated time since baseline as a continuous variable and included patient identifier as a clustering variable to account for within-subject correlation across repeated measurements.^34^ Interaction terms between time and clinical outcome were included to allow microbial trajectories to differ between responders and non-responders. All models were adjusted for age, sex, BMI, cohort, prior antibiotic exposure, and prior chemotherapy. Species with low prevalence across samples were excluded to improve model stability. Statistical inference focused on differences in estimated longitudinal trajectories between outcome groups, rather than on cross-sectional contrasts at individual time points. To assess the robustness of longitudinal associations and account for potential clinical heterogeneity, we performed additional sensitivity analyses using the same modeling framework. Specifically, analyses were repeated (i) in a restricted cohort excluding deceased patients (survivor cohort, n = 26), and (ii) in a clinically homogeneous subgroup of patients with chronic lymphocytic leukemia (n = 15). Model specification, covariate adjustment, and inference procedures were identical to those used in the primary analysis. Results from these sensitivity analyses were compared qualitatively to the full cohort to assess consistency of key microbial associations.

To derive a parsimonious classification tool, we developed a predictive model using a fast-and-frugal tree (FFT) to classify patients into risk groups, as previously described in microbiome studies.^17^^,^^35^ FFTs are simplified decision tree models that prioritize interpretability by relying on a limited number of key features, while maintaining competitive performance compared with more complex approaches such as random forests. This approach was chosen to balance interpretability and robustness in the context of a small sample size, and to facilitate biological interpretation by focusing on a concise set of discriminative variables. To evaluate the internal robustness of the FFT classifier, we performed repeated stratified 5-fold cross-validation. Model performance was summarized using the area under the receiver operating characteristic curve (AUC) across resampled test folds. Data were processed using default settings unless otherwise specified.^36^

## 3. Results

### 3.1. Baseline patient characteristics

A total of 291 fecal samples were collected longitudinally from 30 patients receiving ibrutinib therapy at La Roche-sur-Yon Hospital (n = 20) and Nantes University Hospital (n = 10) between February 2020 and May 2023. Patient demographic and clinical characteristics are summarized in Table 1. The median age was 71.5 years (range, 54.0–83.0 years), with 11 females (37%) and 19 males (63%). Twelve patients (40%) received systemic antibiotic treatment during the three months preceding ibrutinib initiation, and four patients (13%) had received chemotherapy prior to starting ibrutinib. During follow-up, four patients (13%) died, and 17 patients (57%) were classified as responders. The estimated progression-free survival (PFS) was 76.7% (95% CI, 62.9–93.4%) at 6 months and 55.6% (95% CI, 40.1–77.0%) at 12 months. The restricted mean PFS was 282 days. Prior chemotherapy exposure was associated with significantly shorter PFS (log-rank test, *p* = 0.034; Supplementary Figure 1), whereas antibiotic exposure before treatment initiation was not (log-rank test, *p* = 0.50; Supplementary Figure 2). Ibrutinib-associated diarrhea was observed in 4 patients (13%) during the first year of ibrutinib therapy.

**Table 1.** Characteristics of patients included in the study who were treated with Ibrutinib.

		Cohorts by center	
	Total cohort	La Roche sur Yon	Nantes
**Characteristic**	**(n = 30)**	**(n = 20)**	**(n = 10)**
**Age (years)**			
Median (range)	71.5 (54.0–83.0)	72.5 (57.0–83.0)	70.5 (54.0–81.0)
≥65, *n* ()	22 (73)	15 (75)	7 (70)
**Gender, *n* (%)**			
Female	11 (37)	8 (40)	3 (30)
**BMI**			
Median (range)	24.8 (18.8–32.6)	24.2 (18.8–32.6)	25.2 (20.8–30.5)
**Medical history, *n* (%)**			
Hypertension	10 (33)	6 (30)	4 (40)
Diabetes	3 (10)	2 (10)	1 (10)
Chronic kidney failure	3 (10)	2 (10)	1 (10)
Chronic obstructive pulmonary disease	5 (17)	4 (20)	1 (10)
Gastro intestinal ulcere	1 (3.3)	0 (0)	1 (10%)
Constipation	2 (6.7)	1 (5.0)	1 (1)
**Diagnosis, *n* (%)**			
Chronic lymphocytic leukemia	15 (50)	9 (45)	6 (60)
Mantle-cell lymphoma	7 (23)	4 (20)	3 (30)
Waldenström macroglobulinemia	8 (27)	7 (35)	1 (10)
**Tabac, *n* (%)**			
Active	4 (13)	4 (20)	0 (0)
Quit	14 (47)	8 (40)	6 (60)
Never smoke	12 (40)	8 (40)	4 (40)
**Antibiotic exposure in the three months period before Ibrutinib administration, *n* (%)**			
No	18 (60)	11 (55)	9 (90)
Yes	12 (40)	9 (45)	1 (10)
**Chemotherapy exposure in the year period before Ibrutinib administration, *n* (%)**			
No	26 (87)	18 (90)	
Yes	4 (13)	2 (10)	2 (20)
**Number of chemotherapy received, *n* (%)**			
One	2 (6.7)	1 (5.0)	1 (10)
Two	1 (3.3)	1 (5.0)	0 (0)
Three	1 (3.3)	0 (0)	1 (10)
**ECOG performance status score, *n* (%)**			
0	21 (70)	13 (65)	8 (80)
1	4 (13)	4 (20)	2 (20)
2	5 (17)	3 (15)	
**Outcome at 12 months, *n* (%)**			
Responders	17 (57)	11 (55)	6 (60)
Nonresponders	13 (43)	9 (45)	4 (40)
**Overall survival, *n* (%)**			
Alive	26 (87)	18 (90)	8 (80)
Death	4 (13)	2 (10)	2 (20)
**Diarrhea during treatment, *n* (%)**			
Yes	4 (13)	3 (15)	1 (10)

### 3.2. Longitudinal changes in gut microbiome diversity during ibrutinib therapy

**Figure 1.**
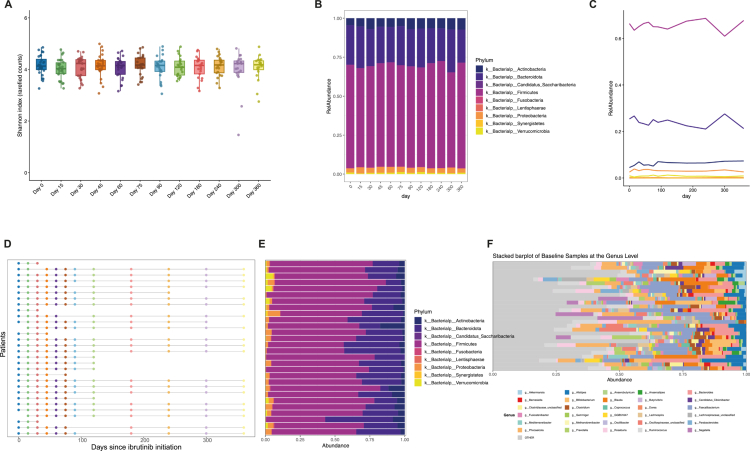
Longitudinal gut microbiome diversity and composition during ibrutinib therapy. **(A)** Bacterial alpha diversity at different time points relative to ibrutinib treatment initiation, calculated using the Shannon index on rarefied counts. **(B)** Relative abundance of bacterial phyla in fecal samples collected at various time points during ibrutinib treatment. **(C)** Temporal variation in the relative abundance of bacterial phyla over the course of ibrutinib therapy. **(D)** Fecal samples collected per patient across study visits. **(E)** Phylum-level relative abundance of baseline fecal samples. **(F)** Genus-level relative abundance of baseline fecal samples.

To characterize microbiome dynamics during ibrutinib treatment, whole-genome shotgun metagenomic sequencing was performed on 291 fecal samples collected longitudinally over one year. Alpha diversity was quantified using the Shannon index across all sampling time points. Compared with baseline (Day 0), microbial alpha diversity remained stable throughout follow-up, with no significant differences detected across time points (Kruskal–Wallis test, *p* = 0.996; all pairwise comparisons and Day 0–based comparisons *p* > 0.60; Figure 1A). At the phylum level, microbial community composition was dominated by Firmicutes and Bacteroidota at all time points (Figure 1B). No significant longitudinal changes were observed for most bacterial phyla during follow-up. Fusobacteriota was the only phylum showing a significant increase over time (*p* = 0.0215), whereas other phyla remained stable (Figure 1C).

**Figure 2.**
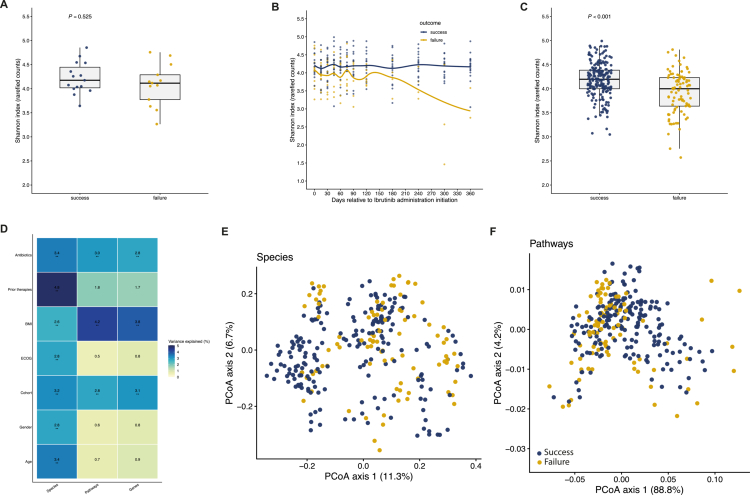
Longitudinal associations between gut microbiome diversity, community structure, and clinical outcome. **(A)** Box plots showing baseline bacterial alpha diversity (Shannon index) in responders and non-responders to ibrutinib treatment. **(B)** Longitudinal variation in microbial alpha diversity over the course of ibrutinib therapy, stratified by clinical response. **(C)** Mean per-patient alpha diversity for samples collected between day 0 (ibrutinib initiation) and day 360, grouped by clinical response. **(D)** PERMANOVA analyses assessing the variance in microbiome features (species composition, metabolic pathways, and microbial gene content) in samples collected between day 0 and day 360. **(E)** Principal coordinate analysis (PCoA) based on Bray–Curtis dissimilarity of microbiome species composition in all longitudinal samples, color-coded by clinical response. **(F)** PCoA based on Bray–Curtis dissimilarity of microbiome pathway composition in all longitudinal samples, color-coded by clinical response.

Baseline microbiome composition was assessed using the fecal sample collected closest to ibrutinib initiation (within −5 to + 2 days; Figure 1D). Baseline samples were available for 28 patients, yielding 1,495 species-level taxa detected in at least one sample. Phylum- and genus-level compositions are shown in Figures 1E and F. While Firmicutes and Bacteroidota predominated across baseline samples, substantial inter-individual variability was observed at the genus level. Baseline alpha diversity did not differ significantly between responders and non-responders (Figure 2A). However, longitudinal mixed-effects modeling revealed a pronounced non-linear temporal pattern of Shannon diversity over follow-up, with a significant effect of spline-modeled time (Type III ANOVA: F = 7.73, *p* = 6.8 × 10⁻⁶). Importantly, the interaction between clinical outcome and time was also highly significant (F = 8.48, *p* = 1.9 × 10⁻⁶), indicating distinct temporal diversity trajectories between responders and non-responders (Figures 2B and C). None of the clinical covariates included in the model—sex, age, body mass index, prior antibiotic exposure, prior chemotherapy, or cohort—were significantly associated with Shannon diversity.

### 3.3. Longitudinal restructuring of microbial community composition

Beta-diversity analysis of baseline samples using Bray–Curtis dissimilarity showed no significant associations with age, sex, body mass index, prior lines of therapy, or antibiotic exposure before ibrutinib initiation (Supplementary Figure 3). Baseline microbial community composition also did not differ significantly between responders and non-responders (Supplementary Figure 4).

In contrast, analysis of all longitudinal samples revealed significant associations between clinical outcome and variance in species composition, metabolic pathway profiles, and microbial gene content (Figure 2D). Homogeneity of dispersion analysis indicated a significant difference in community variability between responders and non-responders (PERMDISP permutation test, *p* = 0.004 for species composition), suggesting that PERMANOVA results may reflect both compositional differences and differences in within-group variability. This difference in dispersion may also be consistent with greater ecological instability of the gut microbiome in patients with unfavorable outcomes. When considering longitudinal trajectories, microbial species composition, pathway abundance, and gene reservoir structure displayed separation according to outcome strata (Figure 2E–F; Supplementary Figures 5–6), independent of major clinical covariates. Longitudinal modeling of principal coordinate axes further supported these findings. While no significant effect of outcome or outcome × time interaction was observed for the first principal coordinate (PC1), analysis of the second principal coordinate (PC2) revealed a significant interaction between outcome and time (*p* = 0.041), indicating divergent temporal community trajectories between responders and non-responders. Age was the only covariate significantly associated with PC2 (*p* = 0.019). These results indicate that clinical outcome is associated with distinct longitudinal remodeling of gut microbiome structure.

### 3.4. Baseline microbial species associated with clinical response

**Figure 3.**
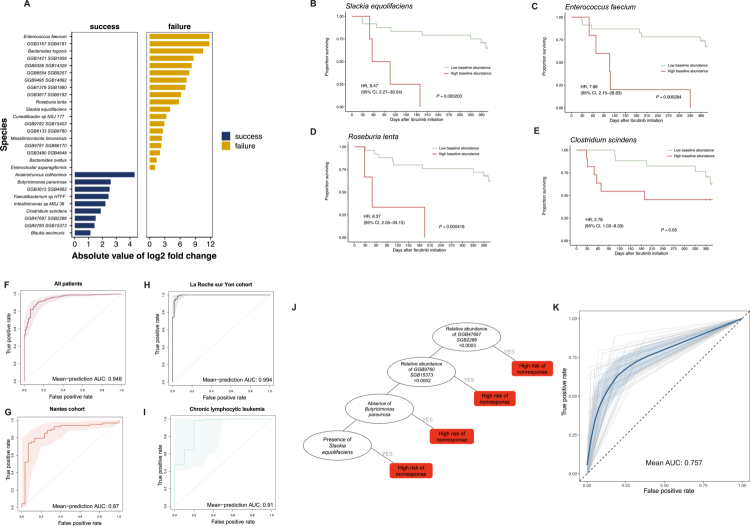
Baseline microbial species associated with clinical response to ibrutinib. **(A)** Differentially abundant bacterial species identified at baseline using ANCOM-BC2, comparing responders and non-responders. Species were filtered for ≥ 5% prevalence, log fold change (LFC) > 1, and false discovery rate (FDR) < 0.05. **(B–E)** Representative Kaplan–Meier survival curves stratified by baseline abundance of selected differentially abundant species, showing differences in progression-free survival according to clinical response. **(F)** Receiver operating characteristic (ROC) curve for outcome classification based on baseline microbial features using tenfold cross-validation with ten repetitions in the full cohort; the shaded area indicates the 95% confidence interval. **(G)** ROC curve for the same classification model applied to patients with chronic lymphocytic leukemia (*n* = 15). **(H)** ROC curve for the same classification model applied to the La Roche-sur-Yon hospital cohort (*n* = 20). **(I)** ROC curve for the same classification model applied to the Nantes University Hospital cohort (*n* = 10). **(J)** Fast-and-frugal tree (FFT)–based decision scheme for outcome classification in patients receiving ibrutinib therapy. **(K)** Fast-and-frugal tree repeated stratified 5−fold cross−validation.

Differential abundance analysis of baseline samples identified bacterial species whose relative abundances differed between responders (n = 15) and non-responders (n = 13). Using ANCOM-BC2 with prevalence filtering (≥5%), a log fold-change threshold >1, and multiplicity corrected with an FDR < 0.05, several species were found to be significantly differentially abundant at baseline (Figure 3A). Species enriched in responders included *Clostridium scindens*, *Blautia caecimuris*, *Enterocloster asparagiformis*, *Anaerotruncus colihominis*, *Butyricimonas paravirosa*, and *Faecalibacterium* sp. HTFF.

Kaplan–Meier analyses stratified by median baseline abundance of these species demonstrated significant associations with progression-free survival (Figure 3B–E). Higher baseline abundances of *Slackia equolifaciens*, *Enterococcus faecium*, *Roseburia lenta*, and *Clostridium scindens* were associated with shorter PFS. A multivariable predictive model based on these discriminating species demonstrated strong classification performance, with an area under the receiver operating characteristic curve (AUC) of 0.95 in the full cohort. Performance remained robust in site-specific analyses, with an AUC of 0.99 in patients from La Roche-sur-Yon and 0.87 in patients from Nantes (Figure 3F–H). To further explore the impact of clinical heterogeneity, we conducted subgroup analyses according to hematological subtype and generated independent ROC curves within each group. Model performance varied across subtypes, with an AUC of 0.91 in chronic lymphocytic leukemia (n = 15), 0.98 in mantle cell lymphoma (n = 7), and 0.60 in Waldenström macroglobulinemia (n = 8). These results suggest that predictive performance may differ according to disease context and highlight the potential influence of underlying clinical heterogeneity (Figure 3I, Supplementary Figure 7).

To derive a parsimonious classification tool, we implemented a stacked machine-learning approach based on fast-and-frugal trees. This approach identified a four-feature decision tree incorporating GGB47687 SGB2286, GGB9760 SGB15373, *Butyricimonas paravirosa*, and *Slackia equolifaciens* (Figure 3J). To assess the internal robustness of the FFT classifier, we performed repeated stratified 5-fold cross-validation. The model achieved a mean AUC of 0.76 (SD 0.05), indicating moderate and reproducible discriminative performance across resampled test sets (Figure 3K).

### 3.5. Longitudinal species- and pathway-level remodeling associated with clinical outcome

We next performed differential abundance analyses on all longitudinal fecal samples collected from the 30 patients during ibrutinib therapy to identify microbial species whose temporal dynamics differed between responders and non-responders. Longitudinal modeling assessed differences in species-level relative abundance trajectories between outcome groups while adjusting for potential confounders, including treatment center, prior antibiotic exposure, and prior chemotherapy. Model parameters were estimated as marginal posterior probability distributions. Post hoc contrasts were computed, and a species was considered differentially abundant between outcome groups if 90% of its posterior distribution did not include zero.

**Figure 4.**
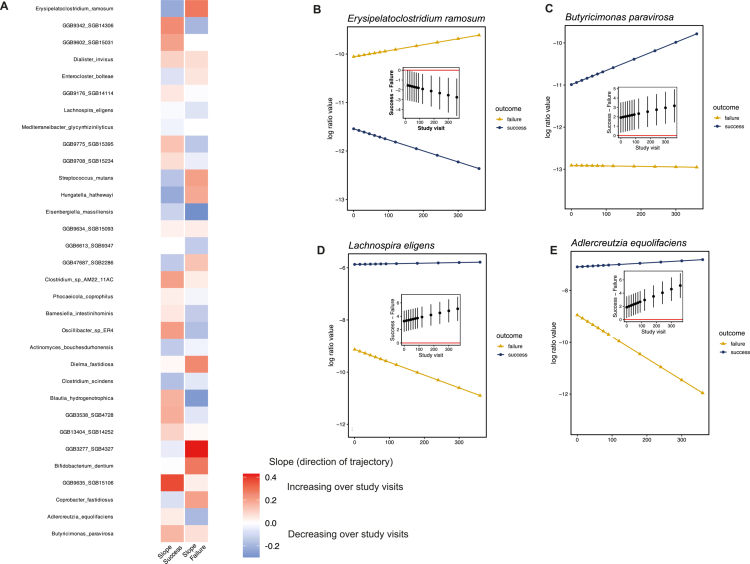
Longitudinal species-level microbiome trajectories associated with clinical response. (A) Overview of longitudinal gut microbiome dynamics in responders and non-responders. For each microbial species shown, slopes indicate whether relative abundance increased or decreased over study visits within each outcome group. Species differentially abundant at only a single study visit were excluded for clarity. Red and blue colors denote increasing and decreasing trajectories, respectively, with color intensity reflecting slope magnitude. (B–E) Representative examples of species-level longitudinal trajectories. Expected relative abundances (centered log-ratio–transformed) are shown over time for responders (blue) and non-responders (yellow). Insets display the mean difference between slopes at each study visit with corresponding 90% credible intervals. Statistical significance was determined based on whether the 90% credible interval excluded zero.

This longitudinal species-level modeling revealed marked divergence in microbial trajectories between patients with successful outcomes and those with nonresponse to ibrutinib treatment, with multiple taxa exhibiting opposing temporal slopes across study visits (Figure 4A). Patients with successful outcomes were characterized by higher relative abundances and/or increasing trajectories over time of several species, including GGB9342 SGB14306, GGB9602 SGB15031, GGB9775 SGB15395, GGB9635 SGB15106, *Clostridium* sp. AM22 11AC, *Oscillibacter* sp. ER4, *Blautia hydrogenotrophica*, *Lachnospira eligens*, *Butyricimonas paravirosa*, and *Adlercreutzia equolifaciens* (Figure 4B–E, Supplementary Table 1). Many of these species, displaying a positive temporal slope exclusively in the responder group, are predominantly saccharolytic and embedded in short-chain fatty acid (SCFA)–associated metabolic networks.

In contrast, patients with nonresponse to treatment exhibited higher relative abundances and/or increasing trajectories of a distinct set of species frequently associated with dysbiosis and inflammatory states, including *Erysipelatoclostridium ramosum*, *Enterocloster bolteae*, *Dialister invisus*, *Streptococcus mutans*, *Hungatella hathewayi*, *Eisenbergiella massiliensis*, *Bifidobacterium dentium*, and *Coprobacter fastidiosus* (Figure 4B−E, Supplementary Table 1). Notably, *Clostridium scindens*, which has been previously associated with pro-inflammatory and tumor-promoting environments, exhibited seemingly discordant patterns across analyses. While it was relatively enriched at baseline in responders, its abundance increased over time in non-responders, contrasting with a declining trajectory in responders. This apparent discrepancy likely reflects differences between cross-sectional and longitudinal analyses and underscores the importance of microbial temporal dynamics, rather than static baseline measurements, in capturing associations with clinical outcomes.

To evaluate the robustness of these findings and the potential impact of clinical heterogeneity, sensitivity analyses were performed in two restricted cohorts: (i) patients who remained alive during follow-up (n = 26), and (ii) patients with chronic lymphocytic leukemia (n = 15). Across these analyses, a proportion of the species identified in the full cohort exhibited consistent directional trajectories and associations with clinical outcome (Supplementary Tables 2–3).

We next evaluated longitudinal changes in microbial metabolic potential by modeling MetaCyc pathway abundances over time. This analysis revealed pronounced differences in temporal trajectories of multiple metabolic pathways between responders and non-responders (Supplementary Figure 8, Supplementary Table 4). Pathways involved in carbohydrate fermentation and short-chain fatty acid–related metabolism, including the Bifidobacterium shunt (P124-PWY) and pyruvate fermentation to propanoate (P108-PWY), exhibited increasing or near-neutral trajectories in responders but declining trajectories in non-responders across follow-up. These pathways are central to the microbial production of acetate and propionate, key SCFAs with known immunomodulatory properties. Conversely, patients with nonresponse demonstrated consistently increasing trajectories for pathways related to amino acid degradation and aromatic compound metabolism, including L-phenylalanine degradation IV (PWY-6318), 4-hydroxyphenylacetate degradation, and L-lysine fermentation to acetate and butanoate (P163-PWY). These trajectories suggest a shift toward proteolytic fermentation, a metabolic profile previously associated with inflammatory gut environments. Marked differences were also observed for pathways involved in bile acid metabolism. Pathways encoding bile acid 7α- and 7β-dehydroxylation (PWY-7754 and PWY-8134) showed strongly positive temporal slopes in non-responders and negative slopes in responders. Finally, several pathways involved in nucleotide biosynthesis and degradation, including purine de novo biosynthesis (DENOVOPURINE2-PWY) and pyrimidine ribonucleoside degradation (PWY-7209), displayed divergent longitudinal trajectories, with increasing trends observed predominantly in the non-responder group. Together, these patterns are consistent with higher microbial turnover or expansion of fast-growing taxa in patients with unfavorable outcomes.

### 3.6. Gut microbiome alterations associated with ibrutinib-associated diarrhea

Associations between gut microbiome dynamics and the development of diarrhea during ibrutinib treatment were next examined. Longitudinal analyses of microbial diversity and pathway abundance revealed patterns broadly similar to those observed for response to treatment (Supplementary Figure 9A–B). Patients who developed diarrhea exhibited distinct longitudinal microbiome trajectories compared with patients who did not, although effect sizes were modest (R^2^ values from 0.002 to 0.015). Baseline differential abundance analysis comparing patients who developed diarrhea during treatment (n = 4) with those who did not (n = 26) identified several species enriched at baseline in patients who remained free of diarrhea. These included *Bacteroides stercoris* (log fold change: 4.90, 95% confidence interval: 3.78 to 6.02), *Blautia massiliensis* (log fold change: 3.09, 95% confidence interval: 2.01 to 4.16), *Coprococcus comes* (log fold change (LFC): 2.29, 95% confidence interval (CI): 1.20 to 3.39), *Dorea longicatena* (LFC: 2.020431, 95% CI: 1.02 to 3.02), and GGB3612 SGB4882 (LFC: 2.89, 95% CI: 1.73 to 4.05, Supplementary Figure 9 C). Many of these taxa are strict anaerobes involved in carbohydrate fermentation and short-chain fatty acid–associated metabolism, suggesting that a more functionally resilient and saccharolytic microbial community may confer protection against digestive toxicity associated by ibrutinib. However, given the very small number of diarrhea cases, these analyses are underpowered and should be considered exploratory and hypothesis-generating, and will require validation in larger cohorts specifically designed to study ibrutinib-associated gastrointestinal toxicity.

## 4. Discussion

The gut microbiome has emerged as a key determinant of clinical outcomes to anticancer therapies, influencing both therapeutic efficacy and treatment-related toxicities, particularly in the setting of immune checkpoint blockade and hematological malignancies.^4^^,^^5^^,^^7^ While multiple studies have demonstrated associations between baseline microbiome composition and treatment response, substantially less is known about how longitudinal microbiome dynamics during therapy shape clinical outcomes. In this study, we performed longitudinal whole-genome shotgun metagenomic sequencing of stool samples collected across multiple study visits in patients treated with ibrutinib, enabling high-resolution, strain- and pathway-level characterization of microbial communities over time. To our knowledge, this represents one of the first longitudinal microbiome studies in this therapeutic context using shotgun metagenomics. Our findings demonstrate that clinical outcomes were not primarily determined by baseline microbial diversity but were instead strongly associated with distinct, outcome-specific microbial trajectories at both the taxonomic and functional levels. Favorable outcomes were characterized by progressive enrichment of saccharolytic, short-chain fatty acid (SCFA)–associated taxa and pathways, whereas nonresponse to treatment was associated with expansion of bile acid–modifying, proteolytic, and inflammation-linked microbial features. Together, these results highlight the importance of microbiome temporal dynamics, rather than static baseline states, in shaping host–treatment interactions and clinical response.

The divergent longitudinal trajectories of microbial diversity observed in our study further underscore the relevance of microbiota recovery during therapy. Patients with successful outcomes exhibited progressive restoration of alpha diversity over time, consistent with recovery of a resilient gut ecosystem, whereas patients with unfavorable outcomes showed persistently reduced diversity and limited recovery. Loss of microbial diversity has been repeatedly linked to impaired colonization resistance, disrupted immune homeostasis, and altered metabolic support to the host, particularly in oncological and hematological settings.^1^^,^^37^^,^^38^ Notably, the early divergence in diversity trajectories suggests that post-treatment microbiome dynamics may serve as an early indicator of host recovery capacity. Although not formally tested in this study, divergence appeared to emerge within the first months of therapy, raising the possibility that early timepoints (e.g., within 2–3 months after treatment initiation) could represent a critical window for identifying patients at risk of unfavorable outcomes and for guiding microbiome-targeted interventions. This hypothesis warrants validation in future prospective studies. These observations support a model in which incomplete restoration of microbial diversity contributes to adverse outcomes and point to the gut microbiome as a potential target for interventions aimed at enhancing ecological resilience in high-risk patients. In addition, prior exposure to chemotherapy was associated with reduced progression-free survival in our cohort and may represent an important confounding factor, as cytotoxic treatments are known to induce long-lasting alterations in gut microbiome composition. Such effects could influence both microbial recovery trajectories and clinical outcomes.

By integrating baseline and longitudinal analyses, our study reveals coordinated species- and pathway-level microbial remodeling associated with response to ibrutinib therapy. Baseline differences between responders and non-responders indicate that pre-treatment gut ecosystem states may influence therapeutic efficacy, with enrichment of anaerobic taxa such as *Butyricimonas paravirosa* and *Faecalibacterium* spp. in responders, suggesting a role for microbial metabolic capacity in shaping treatment outcomes. More striking, however, were the pronounced divergences in microbial trajectories observed during follow-up. Patients with favorable outcomes exhibited increasing or sustained abundances of strict anaerobes including *Lachnospira eligens*, *Butyricimonas paravirosa*, *Blautia hydrogenotrophica*, and *Adlercreutzia equolifaciens*. These taxa are predominantly saccharolytic and embedded within SCFA-associated metabolic networks that support complex carbohydrate fermentation, microbial cross-feeding, and immune homeostasis.^39^ In particular, *L. eligens* and *B. paravirosa* are well-established fiber-responsive SCFA producers,^40^^,^^41^ whereas *A. equolifaciens* mediates isoflavone-to-equol conversion, generating metabolites with documented anti-inflammatory and immunomodulatory properties.^42^ In contrast, patients with nonresponse to treatment showed enrichment of taxa frequently associated with dysbiosis and inflammatory states, including *Erysipelatoclostridium ramosum*, *Enterocloster bolteae*, *Dialister invisus*, *Streptococcus mutans*, *Hungatella hathewayi*, and *Bifidobacterium dentium.*^43–46^ Notably, *Clostridium scindens*, a key carrier of the bile acid–inducible (bai) operon, displayed opposing trajectories between outcome groups, consistent with its established role in secondary bile acid production and pro-inflammatory environments.^47^^,^^48^

Functional pathway analyses closely mirrored these taxonomic patterns. Pathways involved in carbohydrate fermentation and SCFA production, including the Bifidobacterium shunt (P124-PWY) and pyruvate fermentation to propanoate (P108-PWY), showed increasing or stable spline-modeled temporal trajectories in responders, whereas declining trajectories were observed in non-responders, based on regression models incorporating interaction terms between time and clinical outcome. These pathways underpin microbial acetate and propionate production, metabolites with well-established immunomodulatory roles.^49-51^ Conversely, non-responders exhibited progressive enrichment of pathways related to amino acid degradation and aromatic compound catabolism, reflecting a shift toward proteolytic fermentation, a metabolic profile previously associated with inflammatory gut ecosystems.^52-54^ Marked differences were also observed in bile acid metabolism, with selective enrichment of bile acid 7α- and 7β-dehydroxylation pathways (PWY-7754 and PWY-8134) in patients with nonresponse to treatment, indicating preferential secondary bile acid production across study visits.^55^ Collectively, these findings suggest that favorable outcomes are associated with a stable, saccharolytic, and SCFA-oriented microbiome, whereas non response to ibrutinib treatment is marked by expansion of bile acid–modifying and inflammation-associated microbial functions. Although our findings identify associations between microbiome composition and clinical outcomes during ibrutinib therapy, the underlying biological mechanisms remain to be elucidated. One possible explanation involves the immunomodulatory effects of microbial metabolites. Short-chain fatty acid–producing bacteria can influence host immune responses by regulating T-cell differentiation, dendritic cell function, and systemic inflammatory signaling, which may in turn modulate antitumor immunity during BTK inhibition. Conversely, expansion of bile acid–modifying bacteria may alter host immune pathways through bile acid receptor signaling, including FXR and TGR5 pathways, or reshape intestinal metabolic environments that influence immune homeostasis. In addition, microbiota-mediated metabolic processes could potentially influence drug pharmacokinetics or pharmacodynamics through direct microbial metabolism or through host metabolic pathways modulated by microbial metabolites. Further mechanistic studies will be necessary to determine whether such interactions contribute causally to treatment response.

This study has several limitations. First, the cohort size was relatively small (n = 30), and only four patients developed ibrutinib-associated diarrhea, limiting statistical power and the robustness of toxicity-related analyses. In addition, all patients were recruited from two centers within the same geographic region, which may restrict the generalizability of the findings. Although the longitudinal design and dense sampling framework enabled a detailed characterization of microbiome temporal dynamics, these results should be considered exploratory and hypothesis-generating, and will require validation in larger, multicenter cohorts. The limited cohort size increases the risk of both false positive and false negative (findings, particularly in the context of multiple testing and complex longitudinal modeling of microbial features. Given the modest sample size, the model may be prone to overfitting, and its generalizability therefore remains uncertain. Moreover, the microbiome-based classification model was evaluated using internal cross-validation only and was not validated in an independent cohort. External validation in independent datasets will be necessary before considering clinical application.^56^ Second, our analyses were performed at the species and functional pathway levels, consistent with widely used approaches for microbiome-based biomarker discovery. However, this framework does not capture potential strain-level variation within key microbial taxa. As strain-level differences may influence microbial function and host interactions, future studies using strain-resolved metagenomic approaches may provide additional insights into microbiome–treatment relationships. Third, the cohort included patients with different underlying B-cell malignancies, which may have introduced heterogeneity in host–microbiome interactions and clinical responses to ibrutinib. Importantly, the non-responder group represents a clinically heterogeneous population, including patients with disease progression, treatment discontinuation, and death from diverse causes. These distinct clinical trajectories may be associated with different underlying microbiome configurations, potentially blurring outcome-specific microbial signals. Future studies with larger sample sizes should aim to stratify non-response according to its underlying clinical etiology, in order to better delineate outcome-specific microbiome signatures and improve the biological interpretability of observed associations. Fourth, homogeneity of dispersion testing revealed differences in microbiome variability between outcome groups, indicating that PERMANOVA results should be interpreted with caution, as they may reflect both compositional differences and differences in community dispersion. Finally, although antibiotic exposure was included as a covariate in our analyses, other factors known to influence gut microbiome composition—such as proton pump inhibitor use, dietary patterns, and concomitant medications—were not systematically captured and may represent residual confounding.^57^ Importantly, as an observational study, our findings do not allow causal inference, and reverse causality cannot be excluded. Microbiome alterations may reflect underlying clinical status, tumor burden, systemic inflammation, or treatment-related factors such as changes in diet or gastrointestinal physiology, rather than directly driving treatment outcomes. Future studies integrating detailed dietary data, comprehensive medication records, and longitudinal clinical phenotyping will be essential to better disentangle host–microbiome interactions and clarify the causal role of the microbiome in modulating response to ibrutinib.

## 5. Conclusion

Taken together, this study provides a comprehensive longitudinal characterization of gut microbiome dynamics in patients treated with ibrutinib and identifies robust associations between microbial species, functional pathways, and clinical outcomes. By leveraging whole-genome shotgun metagenomics and longitudinal modeling, we demonstrate that treatment response is linked to the expansion of cooperative, saccharolytic, and SCFA-associated microbial ecosystems, whereas non response to ibrutinib treatment is characterized by persistent dysbiosis involving bile acid–modifying, proteolytic, and inflammation-associated taxa and pathways. These findings underscore the importance of microbiome temporal trajectories, rather than static baseline composition, in shaping therapeutic response. Future mechanistic studies using gnotobiotic and preclinical models will be essential to establish causality and to dissect the immune and metabolic pathways through which specific microbes influence treatment efficacy. Ultimately, our results suggest that microbiome-informed stratification and targeted microbiome modulation may represent promising strategies to enhance clinical outcomes and personalize therapy in patients receiving ibrutinib. In conclusion, our findings highlight a potential role of gut microbiome dynamics in modulating response to BTK inhibition and support the need for larger, prospective studies to validate these observations.

## Supplementary Material

Supplementary table 3.docx Supplementary table 3.docx

Supplemental Figure 2.pdf Supplemental Figure 2.pdf

Supplementary table 2.docx Supplementary table 2.docx

Supplemental Figure 4.pdf Supplemental Figure 4.pdf

supplemental figure 5.pdf supplemental figure 5.pdf

Supplementary table 1.docx Supplementary table 1.docx

supplemental figure 3.pdf supplemental figure 3.pdf

Supplemental Figure 1.pdf Supplemental Figure 1.pdf

supplemental figure 6.pdf supplemental figure 6.pdf

Supplementary Figure 7 REDO.pdf Supplementary Figure 7 REDO.pdf

Supplemental Figure 8.pdf Supplemental Figure 8.pdf

Supplemental Figure 9.pdf Supplemental Figure 9.pdf

Suplementary Table 4.docx Suplementary Table 4.docx

Supplementary caption.docx Supplementary caption.docx

## Data Availability

Sequencing data are available in the European Nucleotide Archive database under the accession numbers PRJNA1417473. The anthropometric and other metadata is not possible, as they were not covered by the participants’ informed consent used for the study. However, the corresponding authors will make pseudonymized data available upon reasonable request. All codes and scripts are available on the zenodo repository: 10.5281/zenodo.17368470.
