# Supplementary material for: Longitudinal gut microbiome dynamics are associated with clinical outcome and toxicity during ibrutinib therapy
Source: Gut Microbes. 2026 Apr 19;18(1):2659397. doi: 10.1080/19490976.2026.2659397 (PMC13094205; doi:10.1080/19490976.2026.2659397)
Supplement: Supplementary table 3.docx [file KGMI_A_2659397_SM1134.docx]

| **Species** | **Slope (responders)** | **Slope (non-responders)** |
| --- | --- | --- |
| GGB3345_SGB4425 | 0.0288632754416167 | 1.98069467749863 |
| Lachnospira_eligens | -0.0305602681681986 | -0.212704713763731 |
| Bacteroides_cellulosilyticus | -0.13788984948116 | -0.276807119460489 |
| Butyrivibrio_crossotus | -0.0303969166425722 | 0.672160877608355 |
| Oliverpabstia_intestinalis | 0.113420304057434 | 0.321890251953034 |
| GGB9359_SGB14334 | -0.0886063985970851 | 1.00660753438846 |
| GGB3653_SGB4964 | 0.390003320178452 | -0.632052811271894 |
| Streptococcus_salivarius | 0.24568176193559 | -0.539963623134002 |
| Faecalibacterium_sp_CLA_AA_H233 | 0.342844222814862 | 0.650439166871438 |
| GGB47687_SGB2286 | -0.210070264486941 | -0.326361700107004 |
| GGB4571_SGB6317 | -0.0285500589581797 | 1.18495012313694 |
| GGB4604_SGB6369 | 0.120678765878024 | 1.15870754402586 |
| GGB3892_SGB5290 | 0.053227663032275 | 1.23177419774284 |
| GGB6613_SGB9347 | 0.110488666333005 | 0.0148528000329445 |
| Faecalibacterium_SGB15346 | 0.0711344777632208 | 0.0917467431151925 |
| Bilophila_wadsworthia | -0.169721538573616 | 0.0327293595917871 |
| Bacteroides_thetaiotaomicron | -0.0846833539895983 | -0.207358289288606 |
| GGB33469_SGB15236 | 0.10536834183788 | 1.20329778642687 |
| Blautia_sp_MCC283 | 0.241429147578858 | -0.943848892212988 |
| GGB9602_SGB15028 | 0.146567169024723 | 1.90673392992529 |
| Pseudoflavonifractor_sp_AF19_9AC | 0.0739443159601909 | 0.181472102791999 |
| Bacteroides_ndongoniae | 0.0267516928461324 | 0.873722703259307 |
| Alistipes_ihumii | 0.237488197218405 | 0.0498626550981166 |
| GGB9621_SGB15073 | -0.0125030252370712 | 0.132167565069183 |
| Phocaeicola_vulgatus | 0.0841515337766213 | -0.176072303180936 |
| GGB9529_SGB14929 | -0.0282492046333404 | 0.171963482103121 |
| GGB3175_SGB4191 | -0.0471649938399277 | 0.335967623295289 |
| GGB74463_SGB54347 | -0.0557404926055187 | 0.341006770560963 |
| Marvinbryantia_SGB4691 | 0.0207260819752062 | -0.0589001008790127 |
| GGB9501_SGB14898 | 0.0544711869446649 | 0.293806745401343 |
| Butyricimonas_paravirosa | 0.105601001094214 | -0.0169375141519902 |
