# Supplementary figures and images for: Longitudinal gut microbiome dynamics are associated with clinical outcome and toxicity during ibrutinib therapy
Source: Gut Microbes. 2026 Apr 19;18(1):2659397. doi: 10.1080/19490976.2026.2659397 (PMC13094205; doi:10.1080/19490976.2026.2659397)

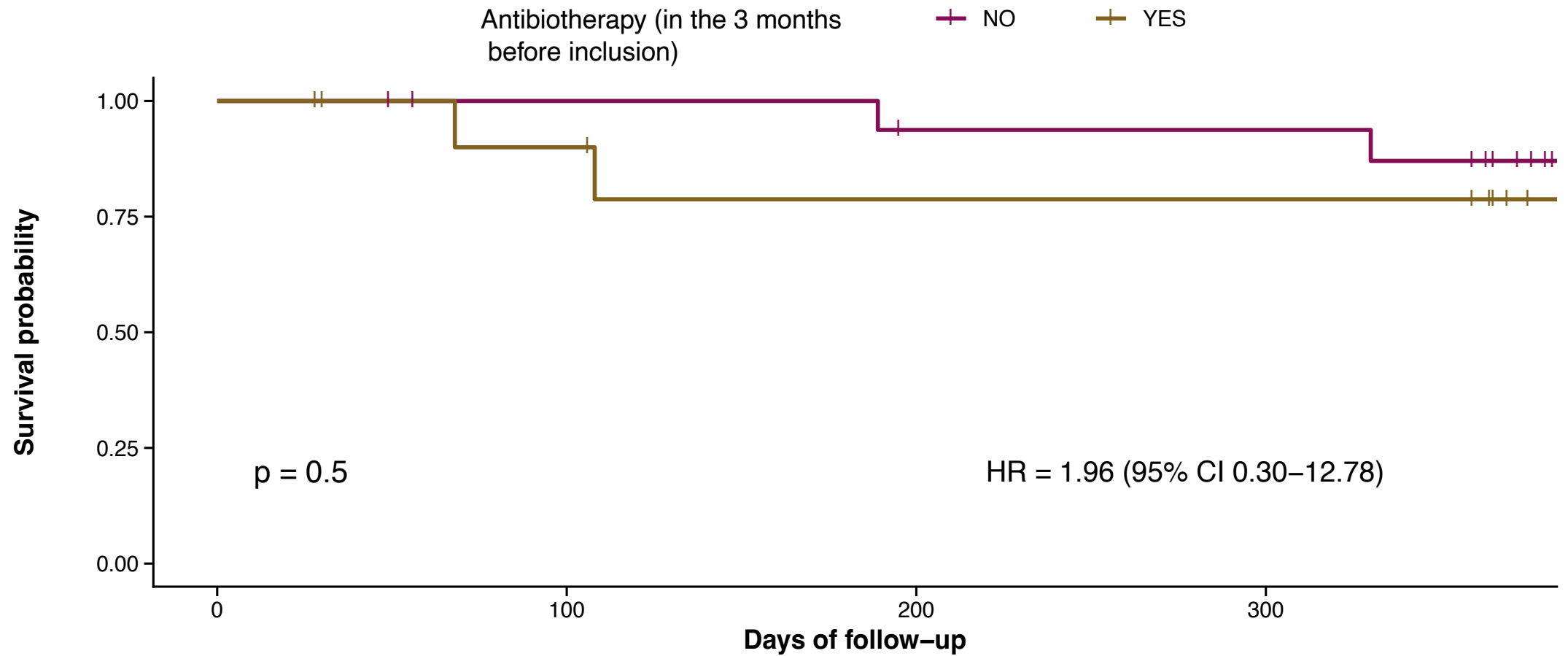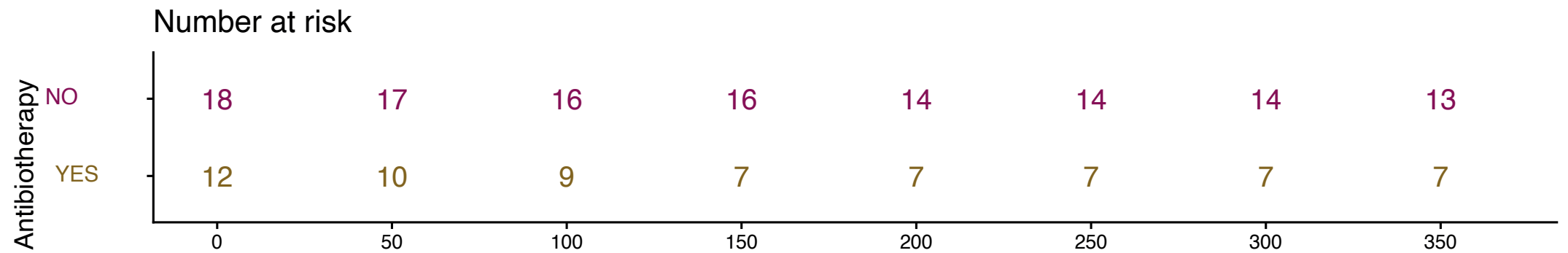

Supplement: Supplemental Figure 2.pdf [file KGMI_A_2659397_SM1122.pdf]

Outcome

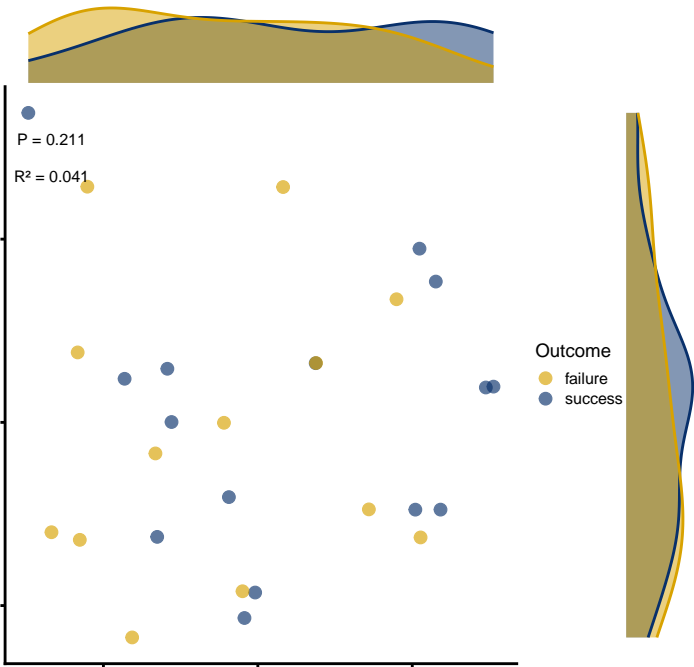

Supplement: Supplemental Figure 4.pdf [file KGMI_A_2659397_SM1124.pdf]

Age

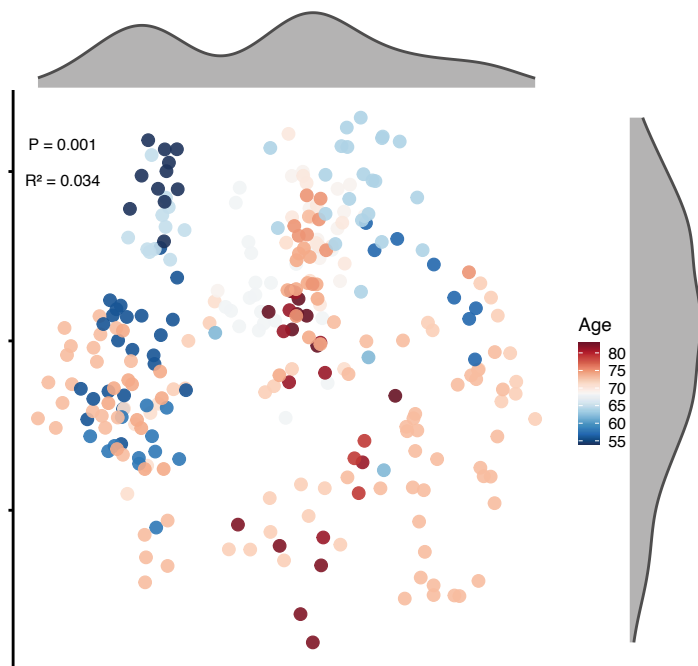

BMI

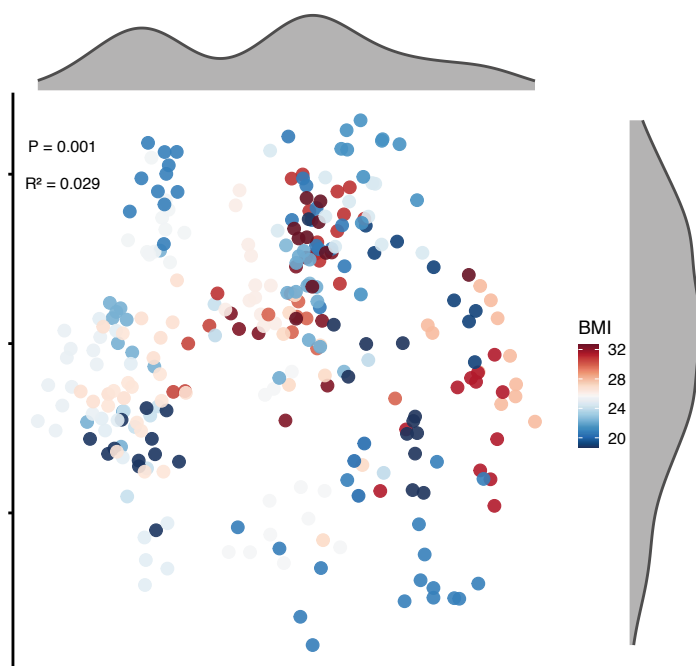Chemotherapy  
before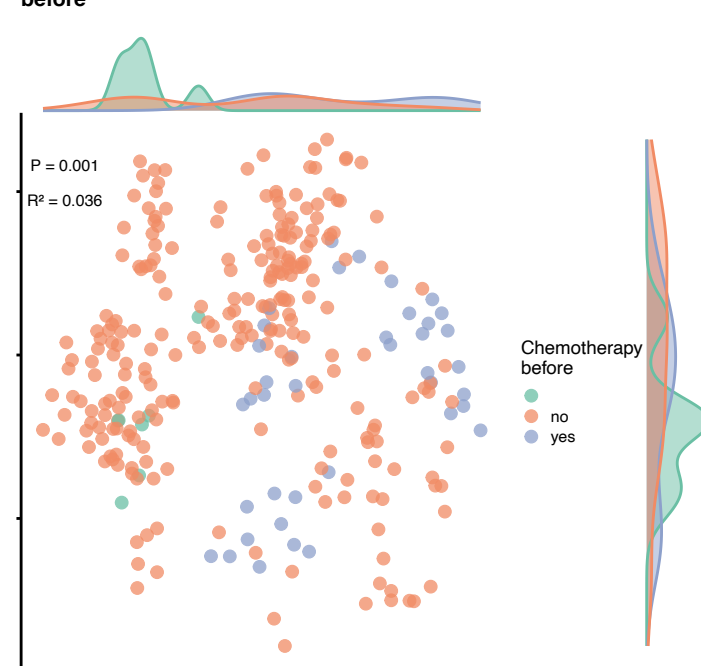

Cohort

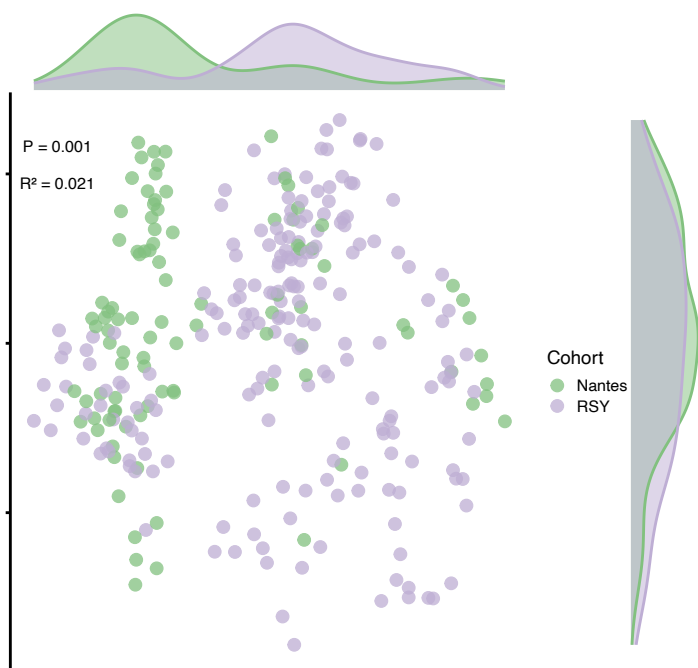

Sex

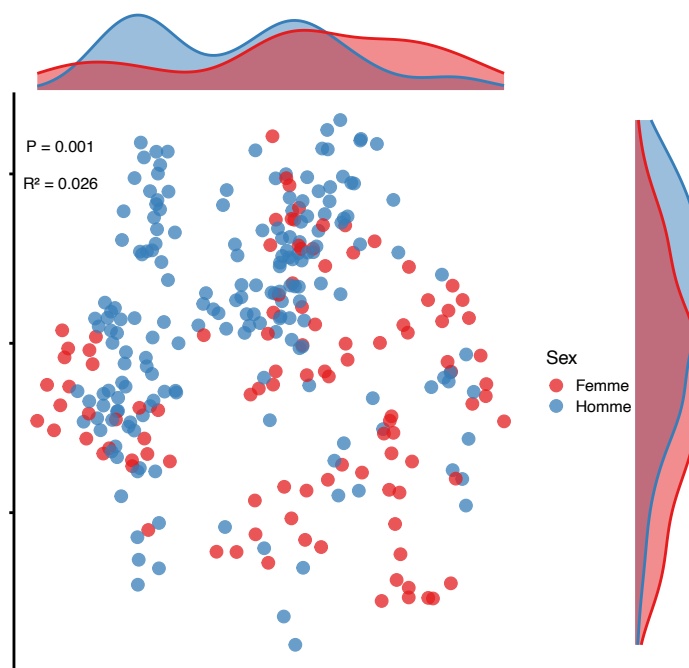Antibiotics  
previous year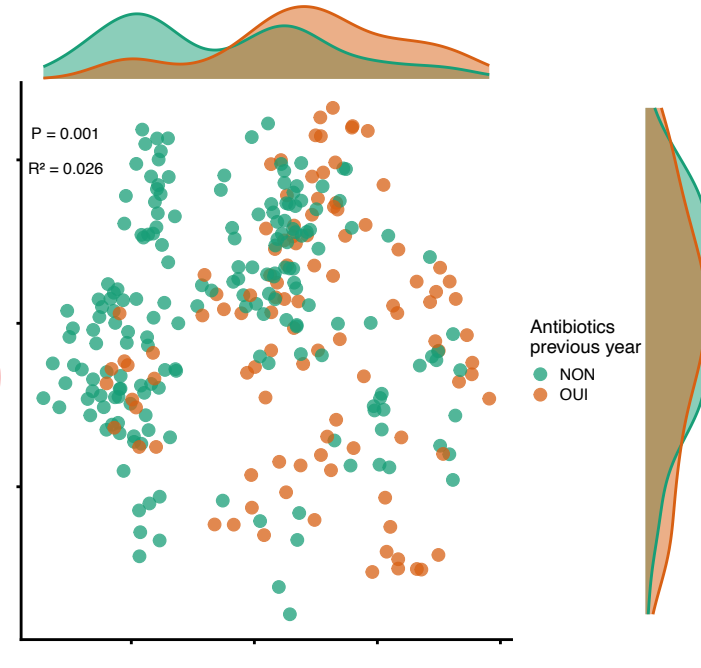

Supplement: supplemental figure 5.pdf [file KGMI_A_2659397_SM1125.pdf]

Age

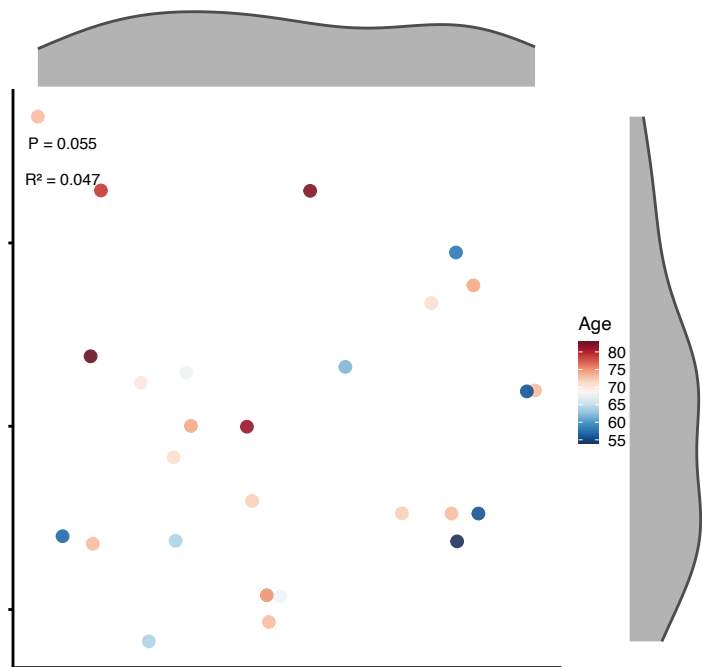

BMI

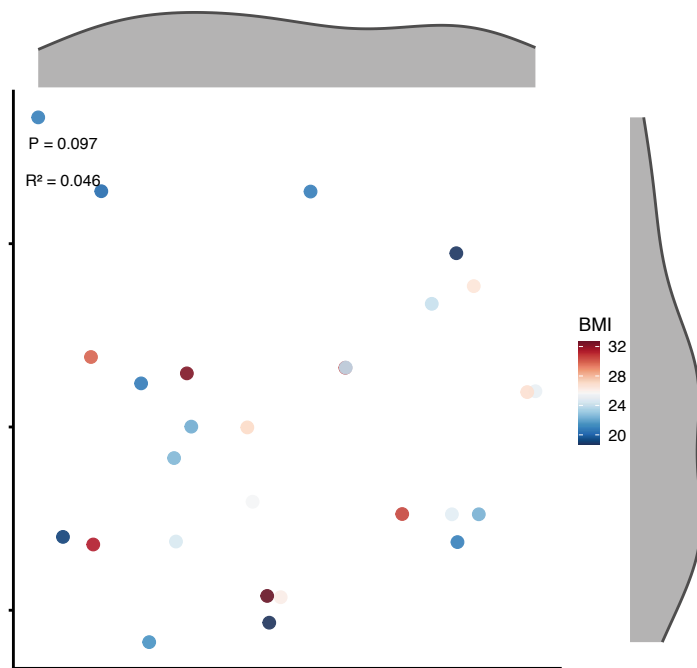Chemotherapy  
before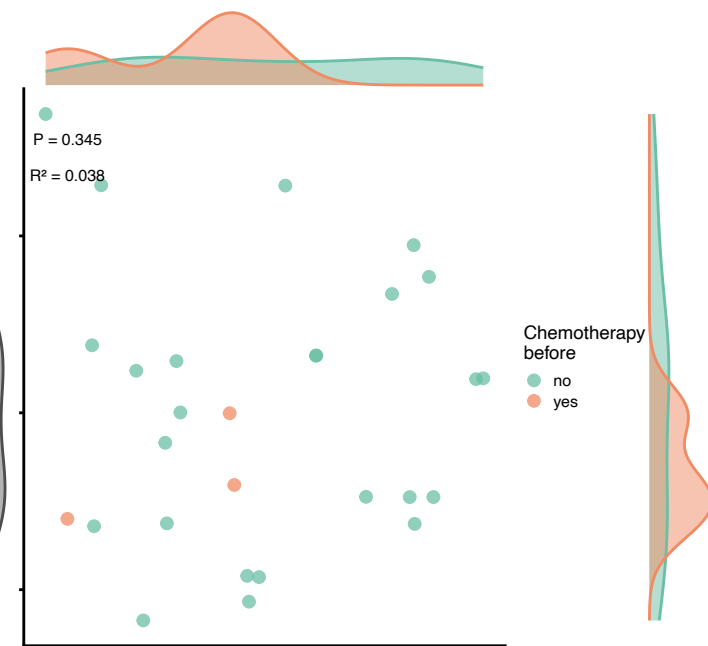

Cohort

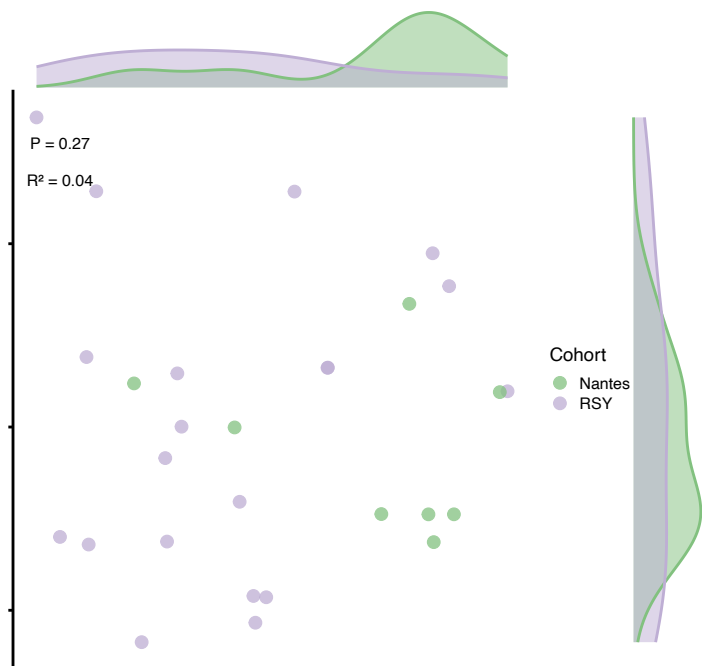

Sex

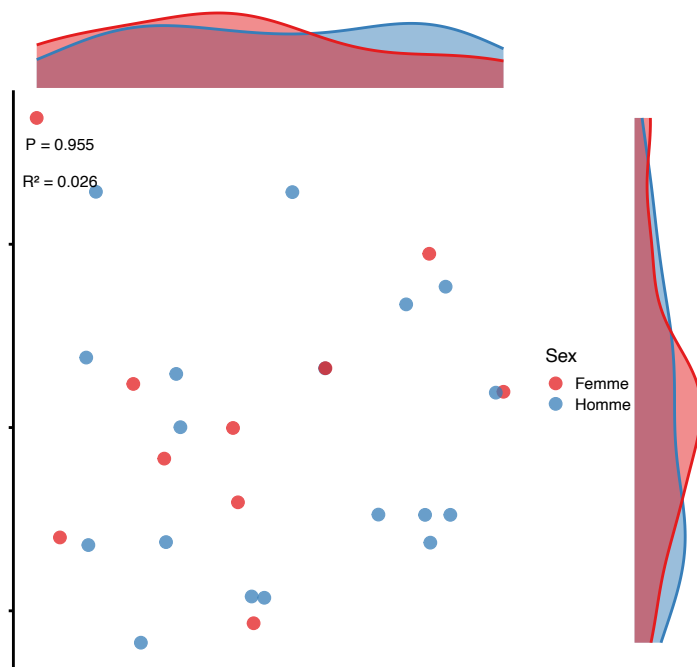Antibiotics  
previous year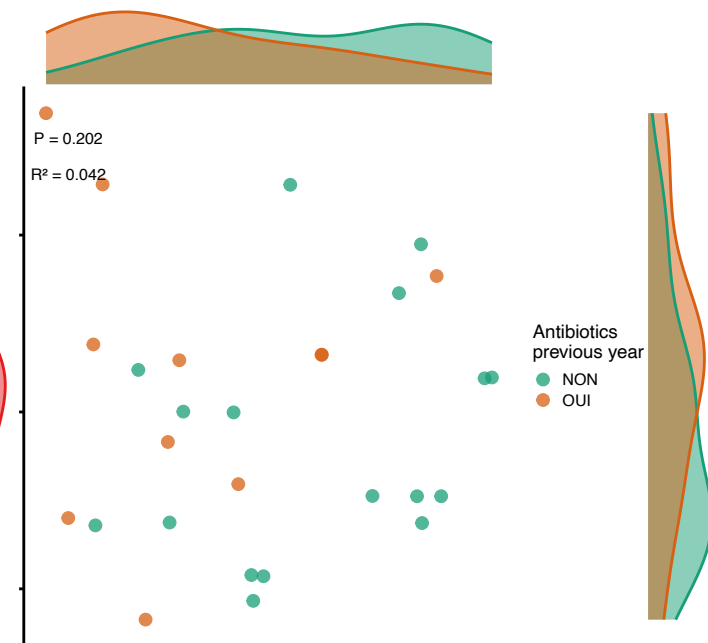

Supplement: supplemental figure 3.pdf [file KGMI_A_2659397_SM1127.pdf]

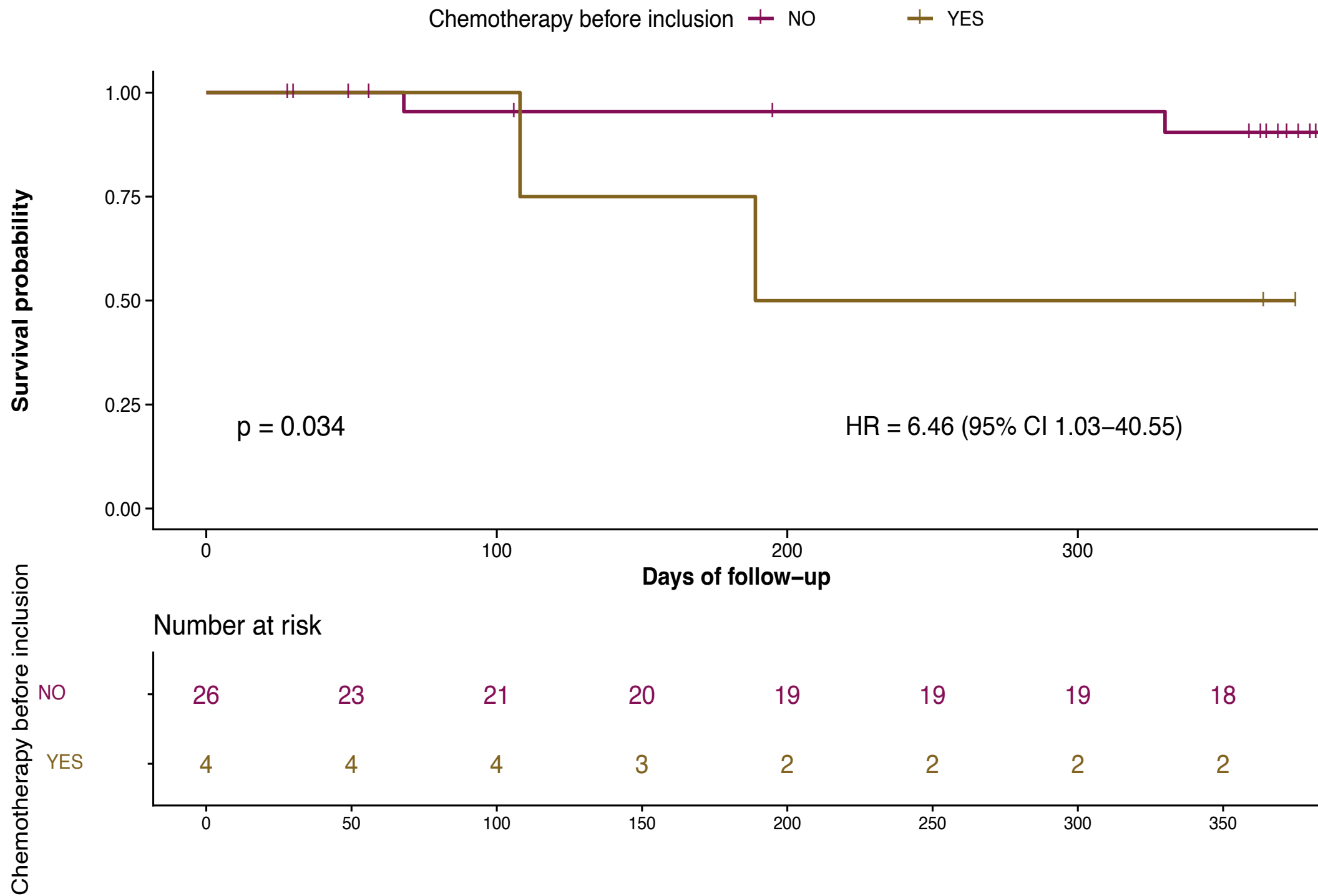

Supplement: Supplemental Figure 1.pdf [file KGMI_A_2659397_SM1128.pdf]

Age

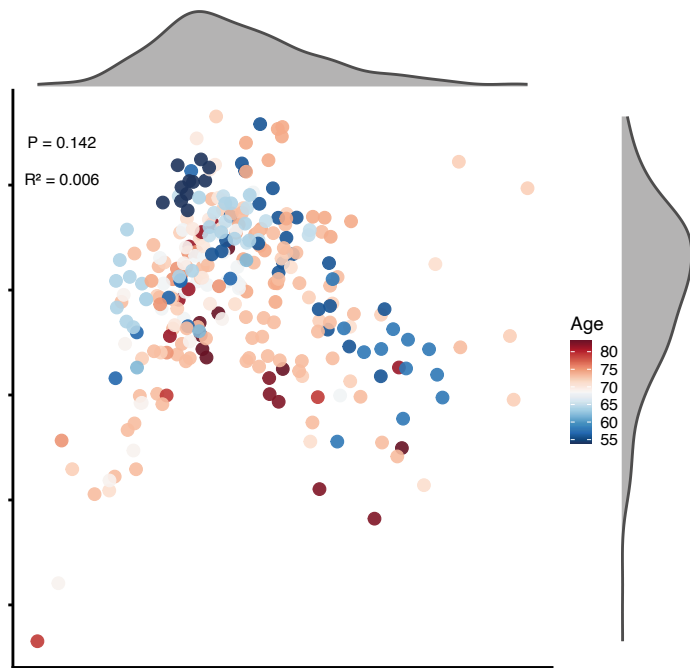

BMI

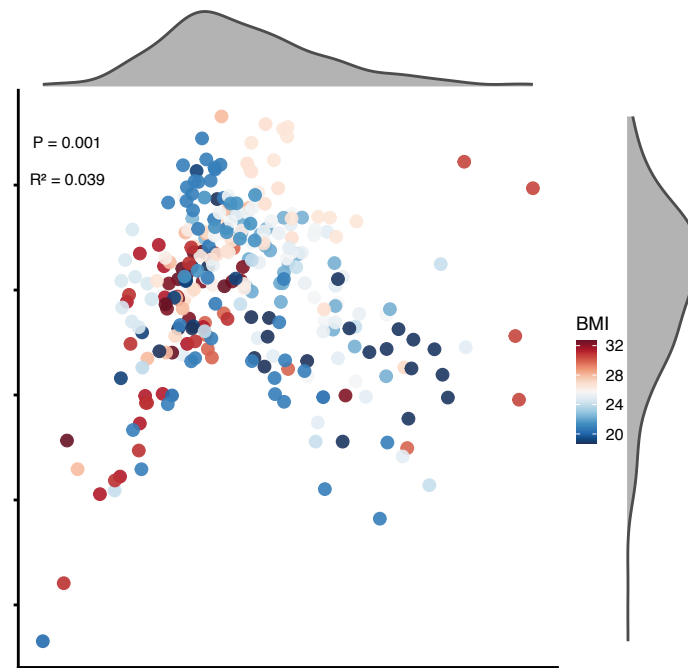Chemotherapy  
before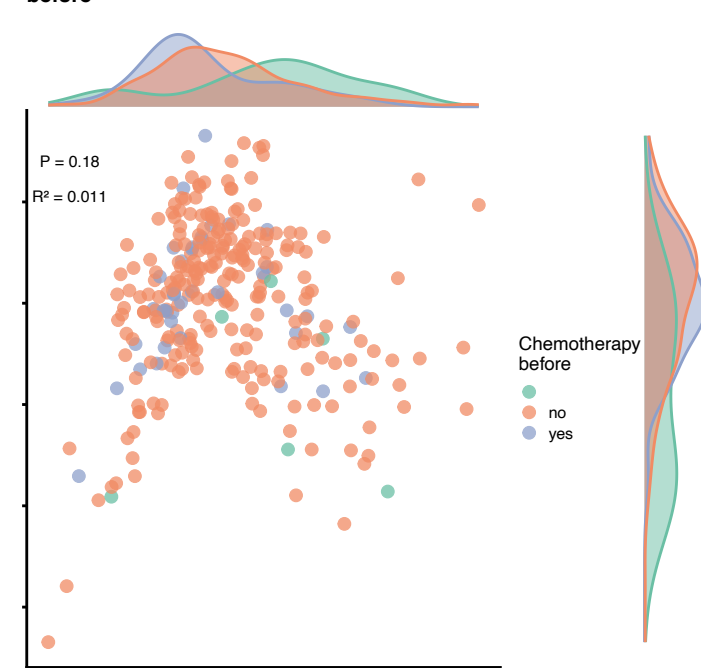

Cohort

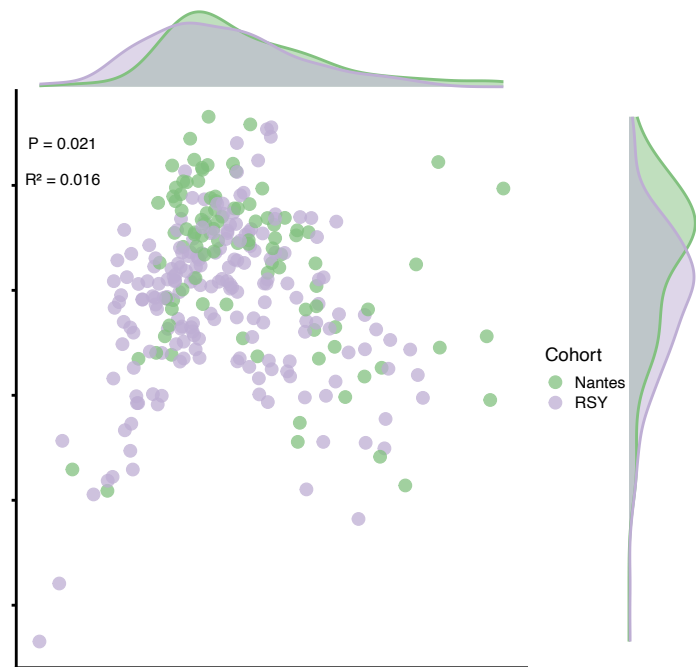

Sex

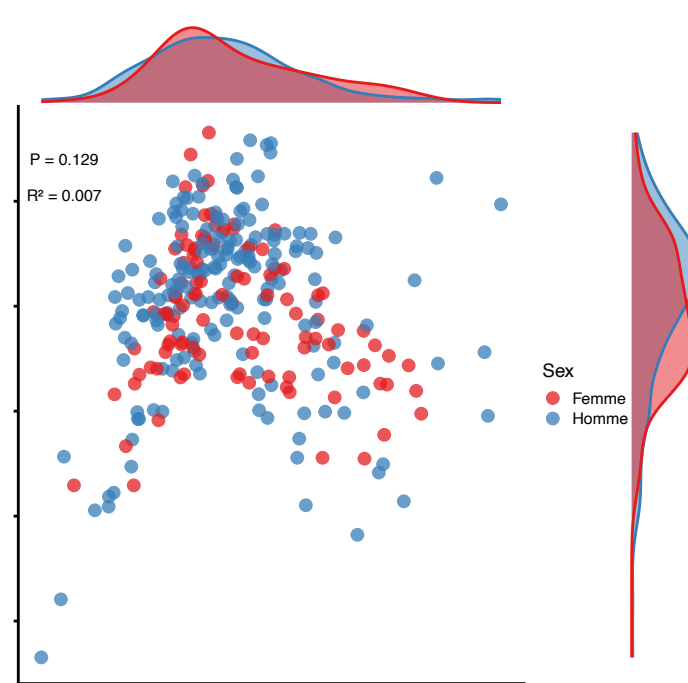Antibiotics  
previous year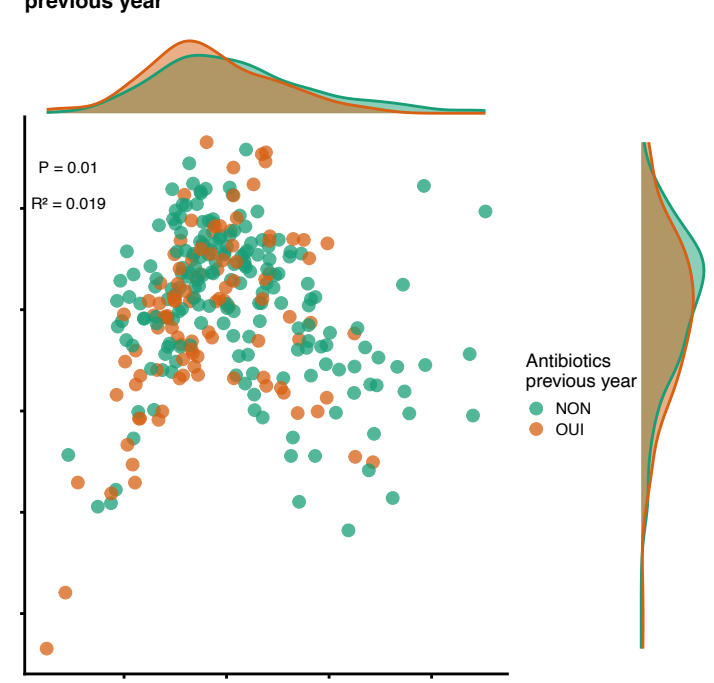

Supplement: supplemental figure 6.pdf [file KGMI_A_2659397_SM1129.pdf]

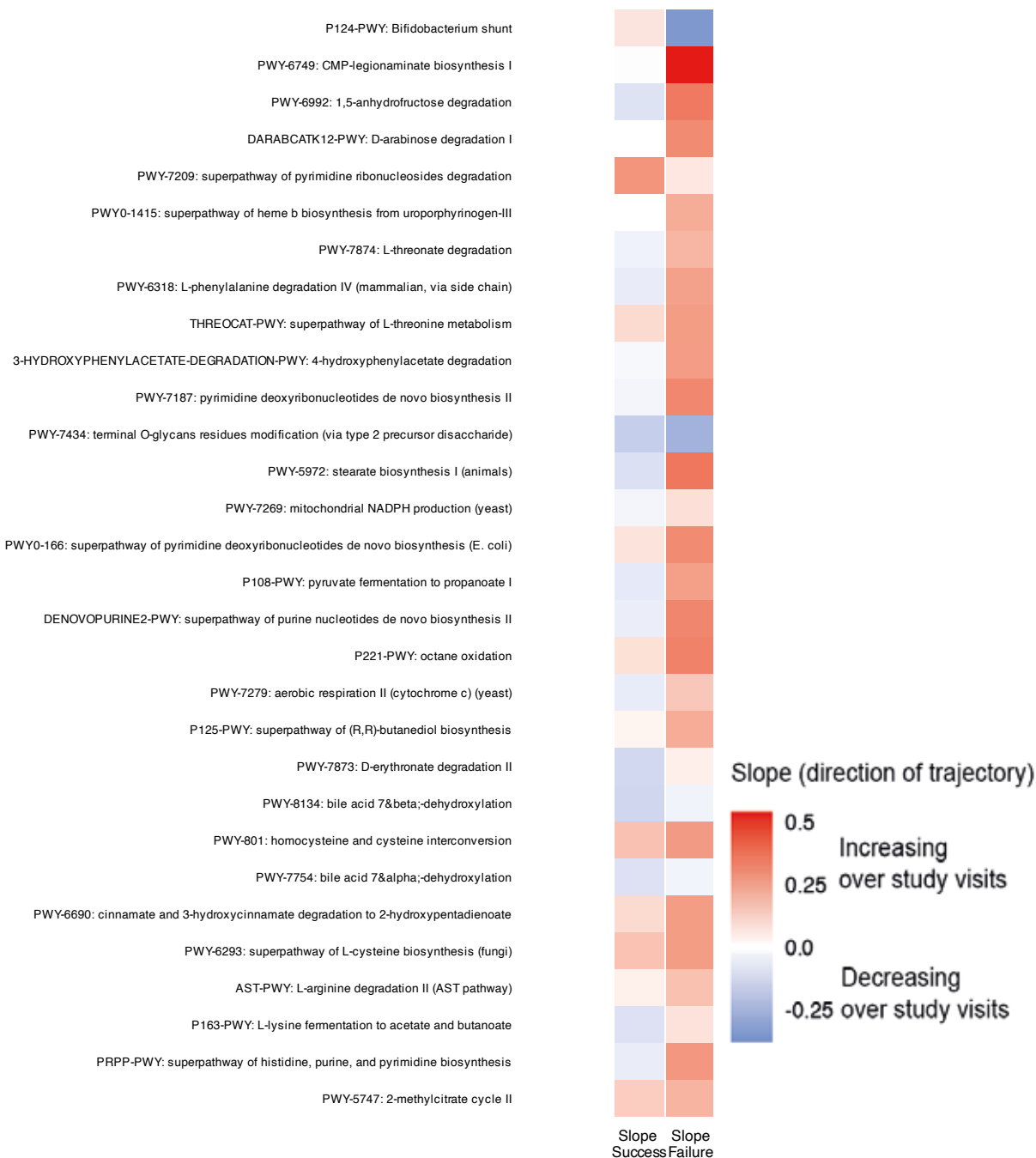

Supplement: Supplemental Figure 8.pdf [file KGMI_A_2659397_SM1131.pdf]

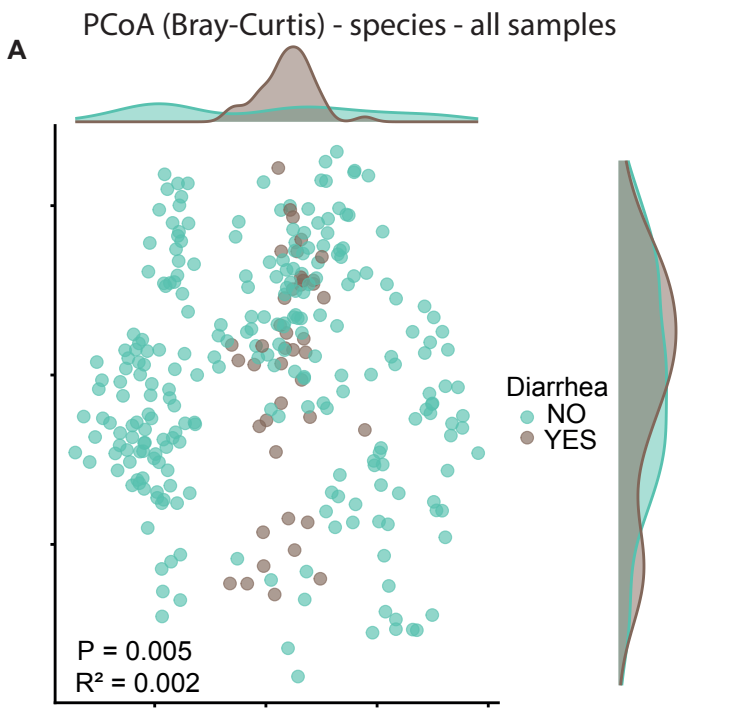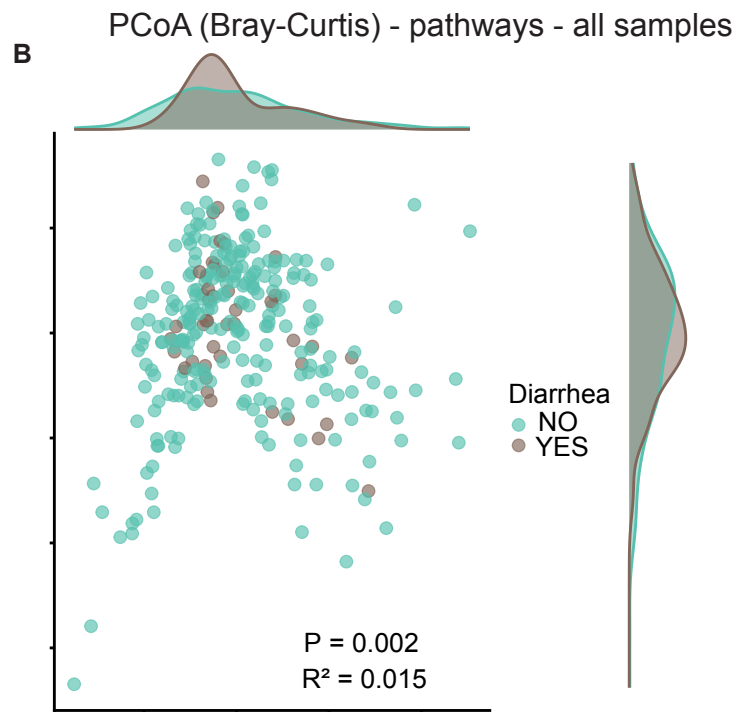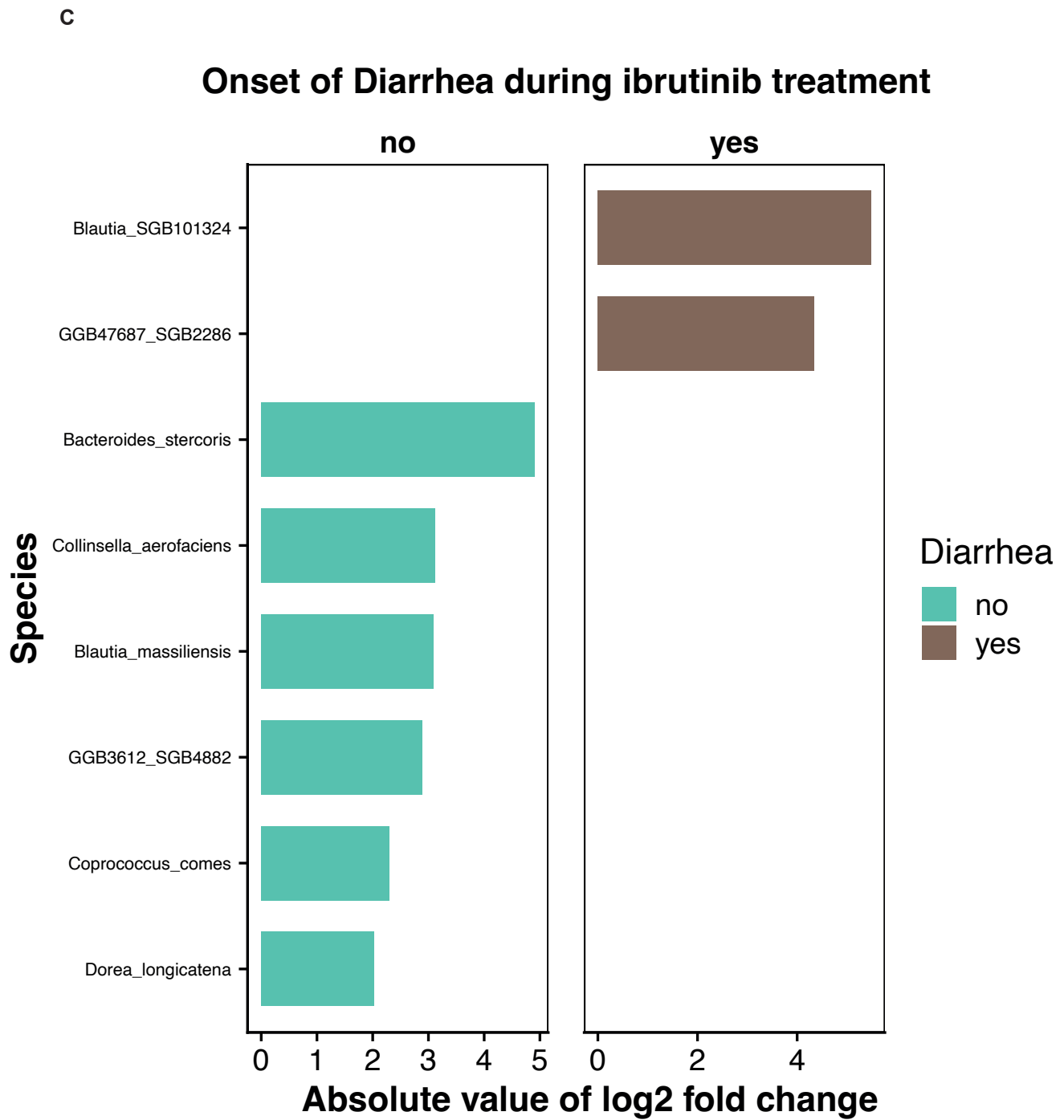

Supplement: Supplemental Figure 9.pdf [file KGMI_A_2659397_SM1132.pdf]
