# Supplementary material for: Longitudinal gut microbiome dynamics are associated with clinical outcome and toxicity during ibrutinib therapy
Source: Gut Microbes. 2026 Apr 19;18(1):2659397. doi: 10.1080/19490976.2026.2659397 (PMC13094205; doi:10.1080/19490976.2026.2659397)
Supplement: Supplementary table 2.docx [file KGMI_A_2659397_SM1123.docx]

| **Species** | **Slope (responders)** | **Slope (non-responders)** |
| --- | --- | --- |
| Dialister_invisus | 0.0924970528160786 | 0.0614573731681341 |
| Lachnospira_eligens | -0.0163808278413487 | 0.0136956562071102 |
| Erysipelatoclostridium_ramosum | -0.22100194745118 | 0.307232002163618 |
| GGB9342_SGB14306 | 0.231777218723465 | -0.234541583059108 |
| Oscillibacter_sp_ER4 | 0.209501844485323 | -0.18958886516192 |
| GGB9602_SGB15031 | 0.192913577557738 | 0.113895771955157 |
| Veillonella_parvula | 0.06797728810169 | 0.561731821079675 |
| Clostridium_sp_AM22_11AC | 0.202126183772285 | -0.154863926644174 |
| Actinomyces_bouchesdurhonensis | -0.147112363963684 | -0.0834046597573012 |
| Enterocloster_bolteae | -0.0744617542943609 | -0.0559480322667896 |
| Barnesiella_intestinihominis | 0.0513781709518369 | 0.0679969839126467 |
| Phascolarctobacterium_succinatutens | 0.00969610592488246 | -0.0411712597963845 |
| GGB3363_SGB4447 | 0.0585277481280427 | 0.151737924570424 |
| GGB9412_SGB14770 | 0.0802352308637099 | -0.0350608443925691 |
| Segatella_copri | 0.145987457247257 | -0.00447846665230497 |
| Longicatena_caecimuris | -0.0484233583342167 | 0.141865291770625 |
| Coprobacter_fastidiosus | -0.0850175376087978 | 0.00988735396743434 |
| GGB9176_SGB14114 | 0.0573988111757797 | -0.004154421703582 |
| Eisenbergiella_massiliensis | -0.0779539213967442 | -0.447797859824495 |
| GGB6613_SGB9347 | -0.00375886703529052 | -0.167103546040425 |
| Blautia_sp_MCC283 | 0.168444815342639 | -0.210611928409605 |
| GGB9635_SGB15106 | 0.362579608404852 | 0.0736141873481706 |
| Faecalibacillus_intestinalis | 0.100268895797384 | -0.030459852866347 |
| Anaerotruncus_massiliensis | -0.161953019951692 | 0.336362500710682 |
| GGB9775_SGB15395 | 0.13271349602959 | -0.0355596797918151 |
| GGB58158_SGB79798 | -0.0768881016566687 | -0.44870062101426 |
| Clostridium_fessum | 0.0619028027639376 | 0.0266605073596816 |
| Pseudoflavonifractor_gallinarum | 0.0156011816158108 | -0.0577767793413634 |
| Bacteroides_cellulosilyticus | -0.146027218249115 | 0.0794055537194974 |
| Sutterella_wadsworthensis | 0.00485333555042441 | -0.0243458412625461 |
| Butyricimonas_paravirosa | 0.15538078134193 | 0.0338862427314052 |
| Adlercreutzia_equolifaciens | 0.0286025075681571 | -0.298129624617878 |
