# Supplementary material for: Longitudinal gut microbiome dynamics are associated with clinical outcome and toxicity during ibrutinib therapy
Source: Gut Microbes. 2026 Apr 19;18(1):2659397. doi: 10.1080/19490976.2026.2659397 (PMC13094205; doi:10.1080/19490976.2026.2659397)
Supplement: Supplementary table 1.docx [file KGMI_A_2659397_SM1126.docx]

| **Species** | **Slope (responders)** | **Slope (non-responders)** |
| --- | --- | --- |
| Erysipelatoclostridium_ramosum | -0.229557094252101 | 0.279394067107791 |
| GGB9342_SGB14306 | 0.241457558092875 | -0.196342437187214 |
| GGB9602_SGB15031 | 0.194659738741931 | -0.0011174164511606 |
| Dialister_invisus | 0.0973738671658023 | 0.0721065793191897 |
| Enterocloster_bolteae | -0.0761679168353927 | 0.0551188633643338 |
| GGB9176_SGB14114 | 0.0497649306683644 | -0.0100041133598367 |
| Lachnospira_eligens | -0.0166705119048871 | -0.0720482488324627 |
| Mediterraneibacter_glycyrrhizinilyticus | -0.0317424274023885 | -0.000467331886243224 |
| GGB9775_SGB15395 | 0.127182863432555 | -0.150903158626784 |
| GGB9708_SGB15234 | 0.0724728116022876 | -0.0452617686741369 |
| Streptococcus_mutans | -0.16218374513046 | 0.199678668434548 |
| Hungatella_hathewayi | -0.228585769997531 | 0.181348592657717 |
| Eisenbergiella_massiliensis | -0.117603111904607 | -0.301840778197292 |
| GGB9634_SGB15093 | 0.0282891104969781 | 0.0382480470194325 |
| GGB6613_SGB9347 | -0.00204323115480931 | -0.139302627921058 |
| GGB47687_SGB2286 | -0.144916603650512 | 0.124880252289629 |
| Clostridium_sp_AM22_11AC | 0.201953458260659 | 0.0420452343750404 |
| Phocaeicola_coprophilus | 0.0340293839484774 | -0.0268511874603637 |
| Barnesiella_intestinihominis | 0.049783361962582 | -0.136800724930305 |
| Oscillibacter_sp_ER4 | 0.206678942641856 | -0.172939398113469 |
| Actinomyces_bouchesdurhonensis | -0.143250470543777 | -0.0328635997589913 |
| Dielma_fastidiosa | 0.0161866512843631 | 0.246874421248848 |
| Clostridium_scindens | -0.171087654046864 | -0.0635514688976144 |
| Blautia_hydrogenotrophica | 0.161525697515792 | -0.276413055682495 |
| GGB3538_SGB4728 | 0.171397468197805 | -0.0688272892186716 |
| GGB13404_SGB14252 | 0.0944195914243066 | 0.0144040826884607 |
| GGB3277_SGB4327 | -0.0502372061499004 | 0.426956747887711 |
| Bifidobacterium_dentium | -0.00705716774136506 | 0.278014236711611 |
| GGB9635_SGB15106 | 0.367706436917048 | 0.0350710398497407 |
| Coprobacter_fastidiosus | -0.0832058854475245 | 0.199519431595235 |
| Butyricimonas_paravirosa | 0.155383078891536 | 0.0663974911703909 |
| Adlercreutzia_equolifaciens | 0.0401888545934579 | -0.188672784273502 |
