# Supplementary material for: Longitudinal gut microbiome dynamics are associated with clinical outcome and toxicity during ibrutinib therapy
Source: Gut Microbes. 2026 Apr 19;18(1):2659397. doi: 10.1080/19490976.2026.2659397 (PMC13094205; doi:10.1080/19490976.2026.2659397)
Supplement: Supplementary Figure 7 REDO.pdf [file KGMI_A_2659397_SM1130.pdf]

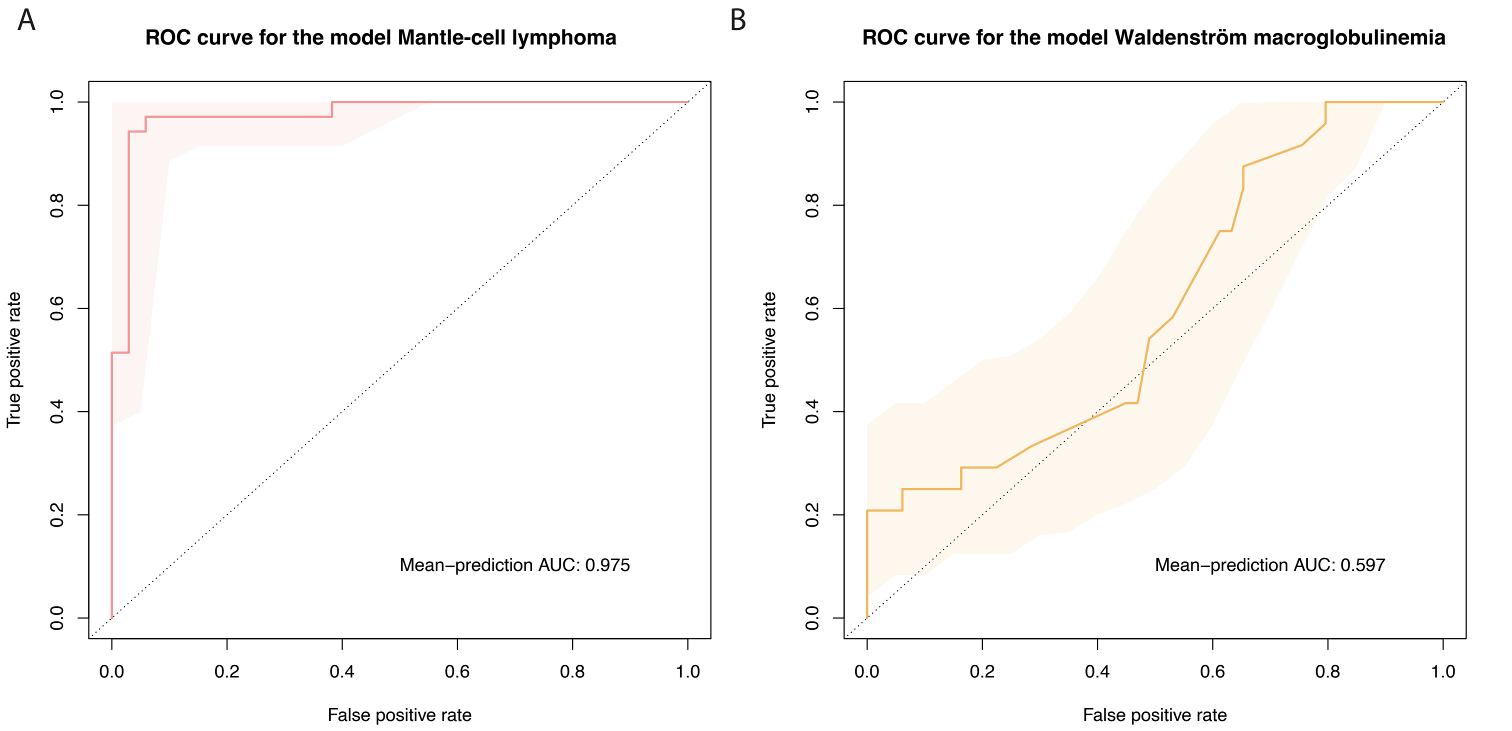

A
ROC curve for the model Mantle-cell lymphoma
True positive rate
False positive rate
0.0
0.2
0.4
0.6
0.8
1.0
Mean–prediction AUC: 0.975
B
ROC curve for the model Waldenström macroglobulinemia
True positive rate
False positive rate
Mean–prediction AUC: 0.597
