# Supplementary material for: Longitudinal gut microbiome dynamics are associated with clinical outcome and toxicity during ibrutinib therapy
Source: Gut Microbes. 2026 Apr 19;18(1):2659397. doi: 10.1080/19490976.2026.2659397 (PMC13094205; doi:10.1080/19490976.2026.2659397)
Supplement: Suplementary Table 4.docx [file KGMI_A_2659397_SM1133.docx]

**Supplementary Table 1. Trajectory slopes for responders vs non-responders of metabolic pathways.**
