# Supplementary material for: Longitudinal gut microbiome dynamics are associated with clinical outcome and toxicity during ibrutinib therapy
Source: Gut Microbes. 2026 Apr 19;18(1):2659397. doi: 10.1080/19490976.2026.2659397 (PMC13094205; doi:10.1080/19490976.2026.2659397)
Supplement: Supplementary caption.docx [file KGMI_A_2659397_SM5876.docx]

**Supplementary Figure legends**

**Supplementary Figure 1.** Progression-free survival (PFS) in the combined cohorts stratified by chemotherapy exposure prior to ibrutinib initiation, assessed up to 12 months following treatment initiation.

**Supplementary Figure 2.** Progression-free survival (PFS) in the combined cohorts stratified by antibiotic exposure during the three months preceding ibrutinib initiation, assessed up to 12 months following treatment initiation.

**Supplementary Figure 3.** Principal coordinate analysis (PCoA) based on Bray–Curtis dissimilarity of microbiome species composition in baseline (Day 0) samples, color-coded by clinical covariates including age, body mass index, prior chemotherapy, cohort, sex, and antibiotic exposure before ibrutinib initiation.

**Supplementary Figure 4.** PCoA based on Bray–Curtis dissimilarity of microbiome species composition in baseline (Day 0) samples, color-coded by clinical response.

**Supplementary Figure 5.** PCoA based on Bray–Curtis dissimilarity of microbiome species composition in all longitudinal samples, color-coded by clinical covariates including age, body mass index, prior chemotherapy, cohort, sex, and antibiotic exposure.

**Supplementary Figure 6.** PCoA based on Bray–Curtis dissimilarity of microbiome pathway composition in all longitudinal samples, color-coded by clinical covariates including age, body mass index, prior chemotherapy, cohort, sex, and antibiotic exposure.

**Supplementary Figure 7.** Receiver operating characteristic (ROC) curve for outcome classification based on baseline microbial features using tenfold cross-validation with ten repetitions; the shaded area indicates the 95% confidence interval. (A) In patients with Mantle-cell lymphoma (n=7), (B) In patients with Waldenström macroglobulinemia (n=8).

**Supplementary Figure 8.** Overview of longitudinal gut microbiome dynamics in responders and non-responders to ibrutinib therapy. For each microbial species shown, slopes indicate increasing or decreasing relative abundance over study visits within each outcome group. Color coding and intensity reflect direction and magnitude of temporal change.

**Supplementary Figure 9.**

**(A)** PCoA based on Bray–Curtis dissimilarity of microbiome species composition in all longitudinal samples, color-coded by onset of ibrutinib-associated diarrhea.

**(B)** PCoA based on Bray–Curtis dissimilarity of microbiome pathway composition in all longitudinal samples, color-coded by onset of ibrutinib-associated diarrhea.

**(C)** Differentially abundant bacterial species at baseline associated with the development of ibrutinib-associated diarrhea, identified using ANCOM-BC2 (≥5% prevalence, LFC >1, FDR <0.05).

**Supplementary Table legends**

**Supplementary Table 1.** Trajectory slopes for responders versus non-responders of species.

**Supplementary Table 2.** Trajectory slopes for responders versus non-responders of species in survivors. Species in common with the analysis in all patients are highlighted in green.

**Supplementary Table 3.** Trajectory slopes for responders versus non-responders of species in patients with chronic lymphocytic leukemia. Species in common with the analysis in all patients are highlighted in green.

**Supplementary Table 4.** Trajectory slopes for responders versus non-responders of metabolic pathways.
